# Long-term quality of life and chronic pain after surgical vs. non-operative treatment of rib fractures: systematic review and meta-analysis

**DOI:** 10.3389/fsurg.2026.1774082

**Published:** 2026-03-30

**Authors:** Xiaojiao Zhu, Wenjun Cao, Chuan Long, Jianwei Han, Suwei Xu, Yingding Ruan

**Affiliations:** 1 Department of Thoracic Surgery, The First People’s Hospital of Jiande, Jiande, China; 2 Department of Thoracic Surgery, Affiliated Zhongshan Hospital of Dalian University, Dalian, China

**Keywords:** chronic chest wall pain, health-related quality of life, meta-analysis, rib fractures, surgical stabilization of rib fractures

## Abstract

**Background:**

Surgical stabilization of rib fractures (SSRF) is increasingly used, yet its long-term impact on patient-centered outcomes remains uncertain. Evidence regarding health-related quality of life (HRQoL) and chronic chest wall pain after SSRF vs. non-operative management is lacking.

**Methods:**

We conducted a systematic review and meta-analysis of studies evaluating SSRF and non-operative treatment in adults with traumatic rib fractures. Primary outcomes were long-term HRQoL (≥3 months) and chronic chest wall pain. Secondary outcomes included tracheostomy. Effect sizes were pooled as standardized mean differences (SMDs), risk ratios (RRs), or mean differences (MDs) with 95% confidence intervals (CIs).

**Results:**

Fourteen studies involving 1,947 patients met the inclusion criteria. Seven studies (*n* = 670; 334 SSRF, 336 non-operative) reported HRQoL, showing no significant difference between groups (SMD 0.10, 95% CI −0.38 to 0.57, *p* = 0.69, I^2^ = 89%). Five studies (SSRF *n* = 213; non-operative *n* = 912) reported chronic pain ≥3 months, with a higher risk after SSRF (RR 1.28, 95% CI 1.03–1.58). Four studies assessing continuous pain scores showed no significant difference. Tracheostomy rates did not differ significantly between groups.

**Conclusions:**

SSRF has no demonstrated long-term HRQoL benefit and may be associated with more chronic chest wall pain than non-operative management. Prospective studies with standardized long-term assessments are needed.

**Systematic Review Registration:**

PROSPERO CRD420251245598.

## Introduction

Traumatic rib fractures are common injuries accounting for approximately 10% of trauma admissions and are frequently associated with significant morbidity, including respiratory compromise, prolonged pain, and impaired functional recovery (1–3). Although most rib fractures are managed non-operatively, a substantial proportion of patients experience persistent chest wall pain, restricted physical activity, and long-term reductions in health-related quality of life (HRQoL) extending months to years after the initial injury (4–7). These patient-centered outcomes are particularly critical because pain-related immobility and respiratory insufficiency contribute to complications such as pulmonary complications, chronic disability, and reduced social reintegration.

Over the past decade, surgical stabilization of rib fractures (SSRF) has gained increasing adoption, driven by evidence showing improvements in short-term physiological outcomes, including improved early respiratory mechanics and reduced need for ventilatory support in selected patients with flail chest or severe displacement (8–12). However, despite growing enthusiasm, the long-term consequences of SSRF remain poorly defined. Many patients continue to report chronic chest wall pain, neuropathic symptoms, or HRQoL impairment regardless of initial treatment, raising uncertainty as to whether the benefits of operative fixation extend beyond the acute recovery phase (5, 6, 13, 14). Moreover, chronic pain after thoracic trauma is multifactorial, potentially influenced by nerve injury, surgical scarring, implant-related discomfort, and altered chest wall mechanics, making the net impact of SSRF on long-term outcomes complex and controversial.

Existing studies evaluating long-term HRQoL or chronic pain after SSRF vs. non-operative care are heterogeneous in design, patient selection, fracture patterns, analgesic protocols, and follow-up duration. Large randomized trials often prioritize short-term clinical endpoints and rarely report extended patient-reported outcomes. Conversely, available observational cohorts vary widely in methodology, with inconsistent assessments of HRQoL instruments and pain definitions, limiting the interpretability and comparability of findings (15–20). As a result, whether SSRF improves, worsens, or has neutral effects on long-term patient-centered outcomes remains uncertain.

In this review, we defined “long-term” as outcomes assessed at ≥3 months after injury, capturing recovery beyond the acute hospitalization and early healing period. Because HRQoL after rib fractures generally improves over time regardless of treatment, any treatment-related differences may be most apparent during the first year and may diminish with longer follow-up as cohorts converge. Accordingly, when studies reported multiple time points, we extracted all eligible follow-up data and prioritized the longest assessment ≥3 months for the primary analyses, while noting time-specific findings (e.g., around 6 months and ≥12 months) when available.

To address this critical evidence gap, we conducted a systematic review and meta-analysis to evaluate long-term (≥3 months after injury) HRQoL and chronic chest wall pain after SSRF compared with non-operative management in adults with traumatic rib fractures. By synthesizing data across randomized trials and cohort studies, this analysis aims to clarify the long-term therapeutic value of SSRF, provide evidence to guide clinical decision-making, and identify priorities for future prospective research. We hypothesized that SSRF would be associated with improved HRQoL and a lower risk of chronic chest wall pain at earlier follow-up (approximately 6 months), with any differences attenuating at longer follow-up.

## Methods

This systematic review and meta-analysis was prospectively registered in the PROSPERO International Register of Systematic Reviews (CRD420251245598) and conducted in accordance with the Preferred Reporting Items for Systematic Reviews and Meta-Analyses (PRISMA) 2020 guidelines (21) and the methodological recommendations of the Cochrane Handbook for Systematic Reviews of Interventions (22). All methodological steps—including literature search, study screening, data extraction and quality assessment—were prespecified and independently performed by two reviewers to ensure methodological rigour and transparency.

### Search strategy

Two investigators independently conducted a systematic search of PubMed, Embase, Scopus, Web of Science Core Collection and the Cochrane Library from database inception to 31 October 2025. The search strategies combined controlled vocabulary (e.g., MeSH and Emtree terms) and free-text keywords related to traumatic rib fractures, SSRF, non-operative management, chronic chest wall pain and HRQoL. No date restrictions were applied; searches were limited to studies published in English. The full electronic search strings for each database are provided in Supplementary Material. In addition, the reference lists of all included articles and relevant reviews were manually screened to identify further eligible studies.

### Inclusion and exclusion criteria

Eligible studies included randomised controlled trials, prospective cohort studies and retrospective cohort studies that compared SSRF with non-operative treatment in adults with traumatic rib fractures and reported at least one prespecified outcome. The primary outcomes of interest were long-term HRQoL (≥3 months after injury) and chronic chest wall pain (reported as either binary presence/absence or continuous pain scores). Secondary outcomes included tracheostomy.

Studies were excluded if they did not provide extractable quantitative data for the outcomes of interest, were case reports, narrative reviews, conference abstracts, animal studies or focused exclusively on non-traumatic rib fractures.

Study selection followed a two-step process, conducted independently by two reviewers. First, duplicate records were removed and titles/abstracts were screened against the eligibility criteria. Second, the full texts of potentially relevant articles were assessed in detail. Any disagreements regarding study inclusion were resolved through discussion, with consultation of a third reviewer when necessary to achieve consensus.

### Data extraction

Data were extracted independently and in duplicate by two reviewers using a standardized, piloted extraction form. The following information was collected from each included study: first author, publication year, country, study design, sample size, patient demographics, fracture characteristics (number and pattern of rib fractures), details of SSRF and non-operative management, HRQoL assessment tools (e.g., EQ-5D, SF-36/SF-12), follow-up duration, chronic pain measures and tracheostomy (secondary outcome).

Health-related quality of life (HRQoL) was extracted as reported in each study using validated generic instruments. For EQ-5D, we extracted the preference-based index (utility) score (and EQ-VAS where reported); for SF-36/SF-12, we extracted the Physical and Mental Component Summary scores (PCS/MCS) when available, or otherwise the overall HRQoL summary measure reported by the authors. We did not recalculate HRQoL from individual questionnaire items. Consistent with standard scoring conventions, higher scores indicate better HRQoL; when necessary, scales were oriented so that higher values reflected better health status. Instrument scoring followed the original developers' algorithms (23, 24).

For studies that reported outcomes at multiple time points (e.g., 3, 6 and 12 months), outcome data were extracted at all reported follow-up time points ≥3 months. For the primary analyses, the longest available follow-up ≥3 months was used; time-specific results (e.g., around 6 months and ≥12 months) were also summarized when available. For chronic pain, both binary outcomes (pain present vs. absent) and continuous pain scores were extracted and analysed as separate endpoints.

Effect size data (means, standard deviations, event counts and risk estimates) were extracted in accordance with the predefined outcome definitions. When continuous outcomes were reported only as medians with interquartile ranges or ranges and no validated method of transformation was feasible, these data were not included in quantitative pooling and were summarised narratively. Newcastle–Ottawa Scale (NOS) scores for observational studies were recorded at the same time. Discrepancies in extracted data were resolved by discussion and, if required, consultation with a third reviewer.

### Assessment of bias

Risk of bias for non-randomised studies was assessed using the NOS, which evaluates study quality across three domains: selection, comparability and outcome/exposure (maximum 9 points). In line with contemporary practice, studies scoring ≥5 points were considered to have at least moderate methodological quality. All assessments were conducted independently by two reviewers, and disagreements were resolved through discussion with a third reviewer.

### Statistical analysis

Meta-analyses were performed using Review Manager (RevMan) version 5.4.1. For continuous outcomes measured on different scales (e.g., HRQoL and pain scores), standardized mean differences (SMDs) with 95% confidence intervals (CIs) were calculated. For continuous outcomes reported on the same scale, mean differences (MDs) with 95% CIs were used. Dichotomous outcomes were summarised as risk ratios (RRs) with 95% CIs.

Given the anticipated clinical and methodological heterogeneity across studies, random-effects models were used as the primary approach for all meta-analyses. Fixed-effect models were applied in prespecified sensitivity analyses when statistical heterogeneity was low (I^2^ ≤ 50%) and when effect estimates were considered sufficiently homogeneous. Statistical heterogeneity was quantified using the I^2^ statistic and Cochran's *Q* test (with *p* < 0.10 indicating significant heterogeneity). Sensitivity analyses were conducted by sequentially excluding individual studies and, where appropriate, by omitting outlier estimates to assess the robustness of the pooled effects.

Publication bias was explored using funnel plots for each pooled analysis. However, because all outcomes included fewer than 10 studies, these plots were interpreted cautiously and considered exploratory. Visual inspection did not reveal clear asymmetry suggestive of major small-study or publication bias.

## Results

### Study selection and overall characteristics

**Figure 1:**
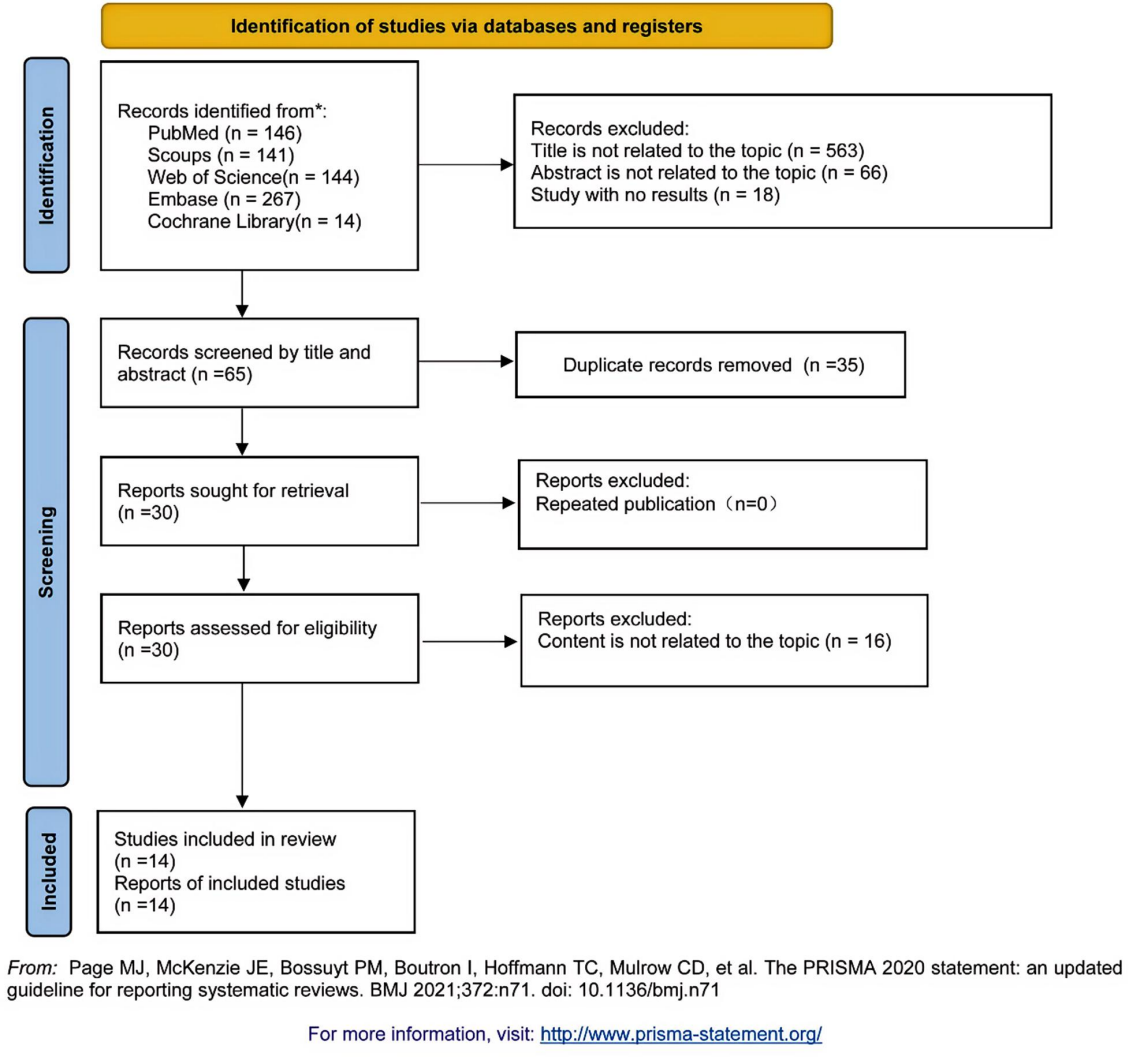
PRISMA 2020 flow diagram summarising identification, screening, eligibility assessment, and inclusion of studies comparing SSRF versus non-operative management for long-term patient-reported outcomes after traumatic rib fractures.

The electronic search from database inception to 31 October 2025 yielded 566 unique records; after de-duplication and a two-phase screening process, 14 studies met the predefined eligibility criteria and were included in the qualitative synthesis (4, 6, 16, 25–35) (Figure 1). These comprised randomised controlled trials as well as prospective and retrospective cohort studies comparing SSRF with non-operative management in adults with traumatic rib fractures. Follow-up durations ranged from 3 months to more than 2 years, and studies varied with respect to injury severity, fracture patterns, indications for SSRF and peri-injury analgesic protocols. Because not all studies reported every outcome of interest, the number of studies and patients contributing to each meta-analysis differed across endpoints. The primary analyses for HRQoL and chronic chest wall pain, together with the secondary outcome of tracheostomy, draw on different subsets of the 14 eligible studies, as detailed in the sections below. Key study and patient characteristics are summarised in Table 1, with detailed treatment characteristics and outcome data provided in Supplementary Table S1. Risk-of-bias assessments for the non-randomised studies using the NOS indicated overall moderate-to-high methodological quality and are presented in Table 2. Reporting of key baseline injury-severity variables, including Injury Severity Score and chest Abbreviated Injury Scale, the presence of flail chest, displacement severity, and bilateral involvement, as well as concomitant injuries, operative indications, and fixation constructs, was inconsistent across studies. This precluded quantitative adjustment for baseline comparability and limited stratified analyses.

**Table 1:** Characteristics of included studies and baseline injury features in adults with traumatic rib fractures.

Study Name	Year	Country	Type of Article	Total Patients (n)	Mean Age (SD)	Male (%)	Surgical Group(n)	Conservative Group(n)	Follow-up Duration （months）
Bauman et al. (25)	2022	USA	Single-center retrospective cohort study with telephone survey follow-up	252	57.3 (15.5) SSRF vs 54.9 (18.8) Non-op	57/78 (73.1%) SSRF vs 113/174 (66.5%) Non-op	78	174	17.3 SSRF vs 20.4 Non-op
Caragounis et al. (26)	2025	Sweden	Bi-institutional prospective observational cohort study	135	59.1 (15.0) Op vs 54.9 (16.1) Non-op	45/62 (72.6%) Op vs 65/73 (89.0%) Non-op	62	73	12 (scheduled follow-up at 6 weeks, 6 months and 12 months)
Farquhar et al. (27)	2016	Canada	Multicenter retrospective cohort study	55	53.1 ± 14.3 Operative vs 56.5 ± 15.9 Nonoperative	15/19 (78.9%) Operative vs 25/36 (69.4%) Nonoperative	19	36	Not provided
Hoepelman et al. (4)	2023	Netherlands and Switzerland (multicenter; majority Netherlands)	Multicenter prospective cohort study	142	63.5 ± 14.7 years Non-operative vs 63.1 ± 13.6 years Rib fixation (after propensity score matching)	56/71 (78.5%) Non-operative vs 55/71 (77.8%) Rib fixation	71	71	12 months (mid-term visit at 6 weeks; telephone follow-up at 12 months; overall 1-year follow-up completion 82%)
Kao et al. (28)	2025	Taiwan	Single-center retrospective cohort study	217	57.62 ± 15.95 years Operative vs 58.41 ± 18.07 years Non-operative	68/103 (66.0%) Operative vs 79/114 (69.3%) Non-operative	103	114	3 (pain scales recorded at 72 h, 2 weeks and 3 months)
Lian et al. (29)	2023	Taiwan	Single-center prospective cohort study	72	57.12 ± 16.50 years Operative vs 55.47 ± 19.28 years Nonoperative	25/34 (73.5%) Operative vs 31/38 (81.6%) Nonoperative	34	38	6 (SF-36 measured at baseline, pre-discharge, 1 month, 3 months and 6 months)
Marasco et al. (16)	2013	Australia	Single-center prospective randomized controlled trial	46 (23 operative vs 23 nonoperative; intention-to-treat)	57.8 ± 17.1 years Operative vs 59.3 ± 10.4 years Nonoperative	20/23 (87.0%) Operative vs 20/23 (87.0%) Nonoperative	23	23	6 (clinical review, CT and spirometry at 3 months; SF-36 quality of life at 6 months)
Marasco et al. (30)	2018	Australia	Single-center retrospective cohort study	1,482	58.9 ± 17.2 years Rib fixation vs 53.4 ± 19.2 years No rib fixation	51/67 (76.1%) Rib fixation vs 1,047/1,415 (74.0%) No rib fixation	67	1,415	24 (telephone follow-up with GOSE, SF-12 and pain NRS at 6, 12 and 24 months)
Marasco et al. (31)	2022	Australia	Multicenter prospective randomized controlled trial	124 (61 operative vs 63 non-operative)	59.1 (15.1) Operative vs 55.0 (15.1) Non-operative	48/61 (77.4%) Operative vs 51/63 (83.6%) Non-operative	61	63	6 (McGill pain and SF-12 at 3 and 6 months)
Meyer et al. (32)	2023	USA	Single-center pragmatic randomized controlled trial	84 (42 Usual care vs 42 SSRF)	49 (15) Usual care vs 50 (15) SSRF	31/42 (74%) Usual care vs 28/42 (67%) SSRF	42	42	6 (EQ-5D-5L at 1, 3 and 6 months)
Prins et al. (6)	2021	Netherlands	Single-center observational cohort study with retrospective data collection and single long-term follow-up (therapeutic level III)	248 (34 SSRF vs 214 Non-op with ≥3 ribs or flail chest; overall cohort *N* = 300)	Median 59 (49–70) SSRF vs 54 (42–63) Non-op; mean not reported	Overall 225/300 (75%) male; distribution by treatment group not reported	34	214	Median 39 (18–65) months after trauma for entire cohort; similar across groups
Walters et al. (33)	2019	United Kingdom	Single-center prospective cohort with retrospective matched nonoperative controls (therapeutic level III)	145 (56 surgical fixation vs 89 nonoperative)	54.2 (16.9) surgical vs 53.4 (17.6) nonoperative	41/56 (73.2%) surgical vs 65/89 (73.0%) nonoperative	56	89	Mean 17.6 (SD 9.5) months surgical vs 20.9 (SD 11.8) months nonoperative; overall mean 18.9 (SD 10.5, range 4–44)
Xu et al. (34)	2025	China	Single-center retrospective observational cohort study (non-flail multiple rib fractures)	124 (61 surgical vs 63 conservative)	58.9 ± 20.0 surgical vs 55.8 ± 19.4 conservative	47/61 (77.0%) surgical vs 46/63 (73.0%) conservative	61	63	Follow-up to 6 months; outpatient visits from 2 to 5 months (median 3 months) with QoL assessed at 3 and 6 months
Zhang et al (35)	2019	China	Single-center retrospective cohort study (severe non-flail chest rib fractures)	78 (39 surgical vs 39 conservative)	48.7 (9.6) surgical vs 50.2 (10.1) conservative	28/39 (71.8%) surgical vs 29/39 (74.4%) conservative	39	39	6 months (pain and SF-36 assessed at 3 and 6 months)

SSRF, surgical stabilization of rib fractures; HRQoL, health-related quality of life; NRS, numerical rating scale; SD, standard deviation; MD, mean difference; SMD, standardised mean difference.

Data are presented as mean (SD) or n/N (%) unless otherwise stated. HRQoL scores and pain NRS are reported at 3, 6 and 12 months after injury when available.Chronic pain outcomes are reported as defined in the original studies. HRQoL was assessed using EQ-5D, SF-12 or SF-36, according to each original study.

**Table 2:** Newcastle–Ottawa scale (NOS) assessment of methodological quality (risk of bias) for non-randomised studies included in this review.

Study	Year	Country	Type of Article	The Newcastle-Ottawa Scale (NOS)		
				Selection	Comparability	Exposure
Bauman et al. (25)	2022	USA	Single-center retrospective cohort study with telephone survey follow-up	***	**	***
Caragounis et al. (26)	2025	Sweden	Bi-institutional prospective observational cohort study	***	**	***
Farquhar et al. (27)	2016	Canada	Multicenter retrospective cohort study	***	***	***
Hoepelman et al. (4)	2023	Netherlands and Switzerland (multicenter; majority Netherlands)	Multicenter prospective cohort study	***	***	***
Kao et al. (28)	2025	Taiwan	Single-center retrospective cohort study	***	**	***
Lian et al. (29)	2023	Taiwan	Single-center prospective cohort study	***	***	***
Marasco et al. (16)	2013	Australia	Single-center prospective randomized controlled trial	***	***	***
Marasco et al. (30)	2018	Australia	Single-center retrospective cohort study	***	**	***
Marasco et al. (31)	2022	Australia	Multicenter prospective randomized controlled trial	***	***	***
Meyer et al. (32)	2023	USA	Single-center pragmatic randomized controlled trial	***	***	***
Prins et al. (6)	2021	Netherlands	Single-center observational cohort study with retrospective data collection and single long-term follow-up (therapeutic level III)	***	***	***
Walters et al. (33)	2019	United Kingdom	Single-center prospective cohort with retrospective matched nonoperative controls (therapeutic level III)	***	***	***
Xu et al. (34)	2025	China	Single-center retrospective observational cohort study (non-flail multiple rib fractures)	***	**	***
Zhang et al. (35)	2019	China	Single-center retrospective cohort study (severe non-flail chest rib fractures)	***	***	***

* Each star (*) represents one point in the Newcastle–Ottawa Scale scoring system.

### Primary outcomes

#### Health-related quality of life (HRQoL)

**Figure 2:**
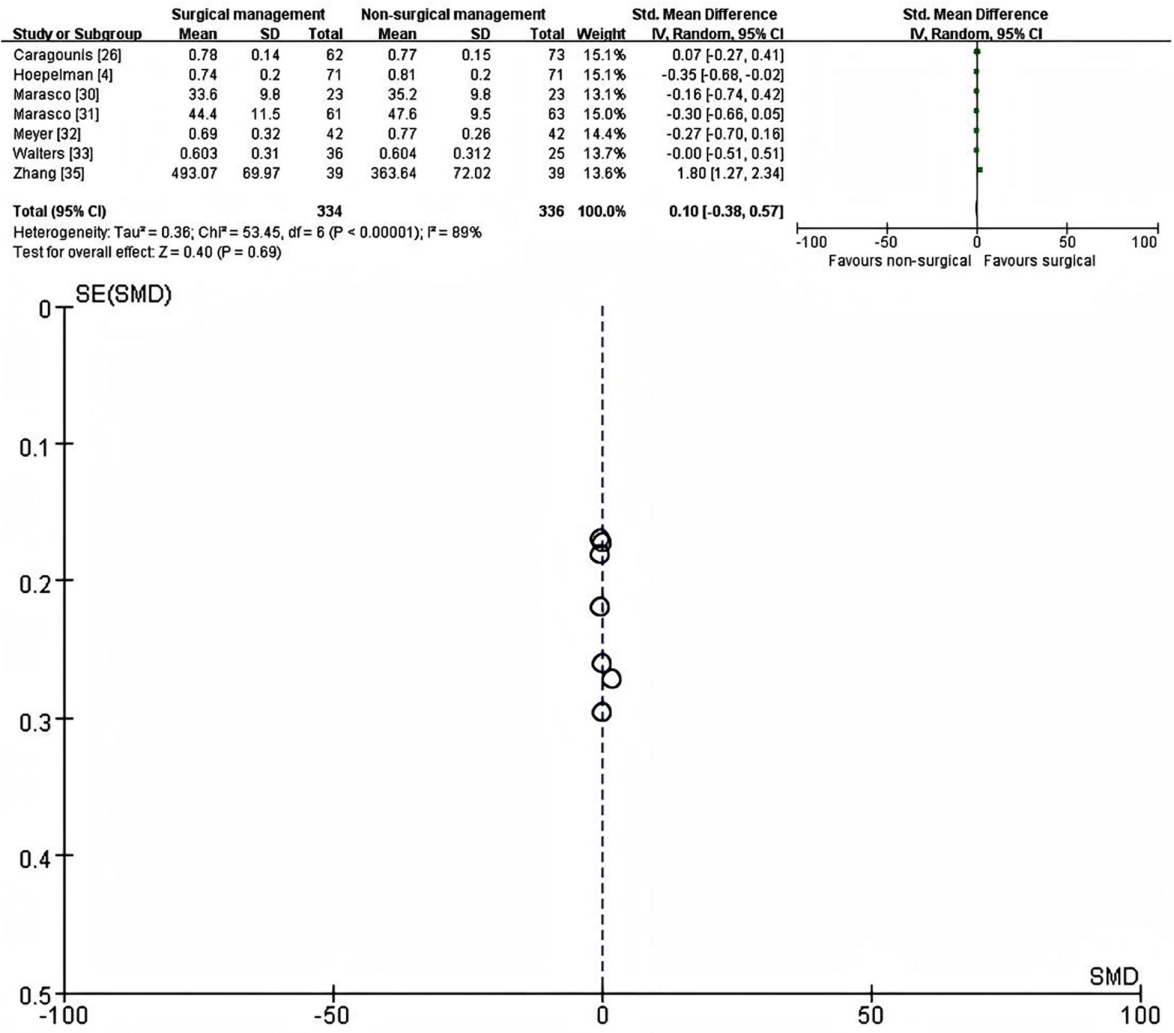
Forest plot (left) and galbraith plot (right) showing the random-effects meta-analysis of standardized mean differences (SMDs) in generic health-related quality of life (HRQoL) at the longest reported follow-up (6–21 months) after surgical stabilisation of rib fractures (SSRF) versus non-operative management. CI, confidence interval; SE, standard error.

Seven studies (*n* = 670; 334 SSRF, 336 non-operative) (4, 24, 30–35) reported long-term HRQoL using validated generic instruments (EQ-5D, SF-36 or SF-12) and were included in the primary meta-analysis (Figure 2). Using a random-effects model with SMD as the effect measure, there was no significant difference in HRQoL at the longest available follow-up (6–21 months) between SSRF and non-operative management (SMD = 0.10, 95% CI −0.38 to 0.57, *p* = 0.69). Statistical heterogeneity was substantial (I^2^ = 89%).

One single-centre Chinese cohort (35) showed an unusually large benefit in favour of SSRF and contributed markedly to heterogeneity. After excluding this outlier, six studies (*n* = 592; 295 SSRF, 297 non-operative) remained. Heterogeneity disappeared (I^2^ = 0%), and the pooled fixed-effect estimate showed a small but statistically significant advantage of non-operative management (SMD = −0.18, 95% CI −0.34 to −0.02, *p* = 0.03) (Supplementary Figure S1). The magnitude of this effect corresponds to a very small standardized difference and is unlikely to be clinically important.

In a prespecified subgroup analysis restricted to studies reporting HRQoL at 6 months, four studies (*n* = 332) were pooled (30–35). The random-effects model again showed no clear difference between groups (SMD = 0.26, 95% CI −0.66 to 1.19, *p* = 0.58) with very high heterogeneity (I^2^ = 94%) (Supplementary Figure S2). After excluding one study (35), three remaining studies (*n* = 254) showed a consistent small benefit of non-operative management (SMD = −0.26, 95% CI −0.51 to −0.02, *p* = 0.04; I^2^ = 0%) (Supplementary Figure S3).

In contrast, among three studies (4, 26, 33) with longer-term follow-up (≥12 months, 12–21 months; *n* = 338) using the EQ-5D index, there was no statistically significant difference in HRQoL between SSRF and non-operative care (fixed-effect SMD = −0.12, 95% CI −0.33 to 0.10, *p* = 0.28; I^2^ = 38%) (Supplementary Figure S4). The direction and size of the effect were consistent with the primary and sensitivity analyses, suggesting that any short-term disadvantage associated with SSRF is small and tends to diminish over time.

### Chronic chest wall pain

#### Binary chronic pain (presence vs. absence)

Five studies reported the presence of chronic chest wall pain at or beyond 3 months after injury (6, 16, 25, 26, 31). For each study, the longest available follow-up was used in the primary analysis: 17–20 months for Bauman (22/78 vs. 48/174) (25), 12 months for Caragounis (4/41 vs. 5/32) (26), 24 months for Marasco (9/16 vs. 191/445) (16), 6 months for Marasco (30/44 vs. 22/47) (31), and ≥12 months for Prins (11/34 vs. 45/214) (6). In total, 213 patients underwent SSRF and 912 were treated non-operatively, with 76/213 (36%) and 311/912 (34%) reporting persistent chest wall pain at the final assessment, respectively.

**Figure 3:**
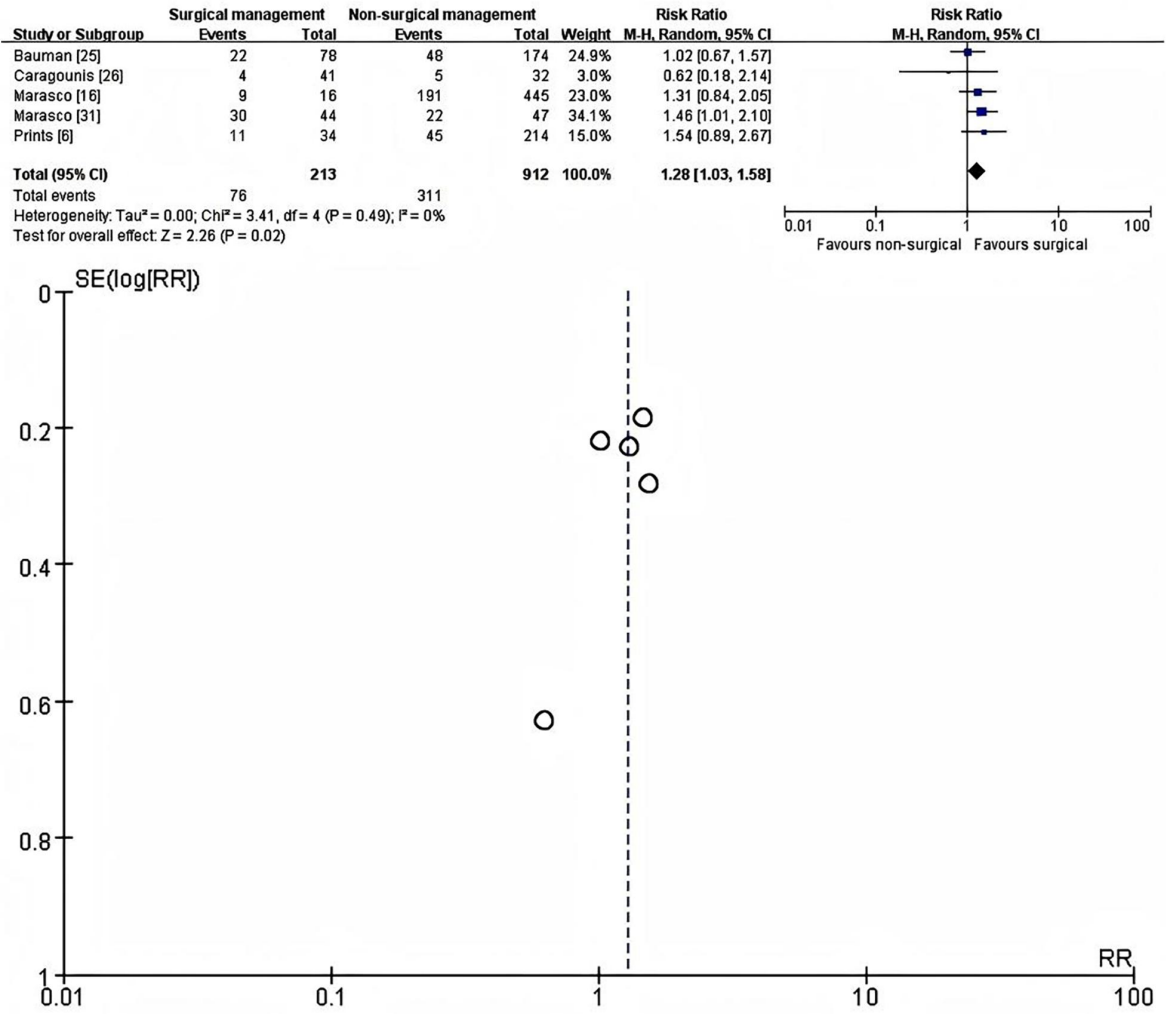
Forest plot (left) and galbraith plot (right) showing the random-effects meta-analysis of binary chronic chest wall pain (present/absent, assessed at >=3 months) after SSRF versus non-operative management. RR, risk ratio; CI, confidence interval; SE, standard error.

Using a random-effects model with risk ratio (RR) as the effect measure, statistical heterogeneity was negligible (I^2^ = 0%, *χ*^2^ = 3.41, *p* = 0.49). SSRF was associated with a higher proportion of patients reporting chronic chest wall pain compared with non-operative management (RR = 1.28, 95% CI 1.03–1.58, *p* = 0.02) (Figure 3). Across individual studies, the prevalence of chronic pain at the longest follow-up ranged from 10% to 68% in the SSRF group and from 16% to 47% in the non-operative group.

#### Continuous chronic pain scores

Four studies reported chronic pain intensity using continuous scales at ≥3 months (27, 28, 33, 35). When more than one post-discharge time point was available, the latest assessment was extracted [Kao (28) and Walters (33) at 3 months; Zhang (35) at 6 months]. The final follow-up means in the SSRF group ranged from 0.36 to 3.41, compared with 0.80 to 3.52 in the non-operative group, on the respective study-specific scales.

**Figure 4:**
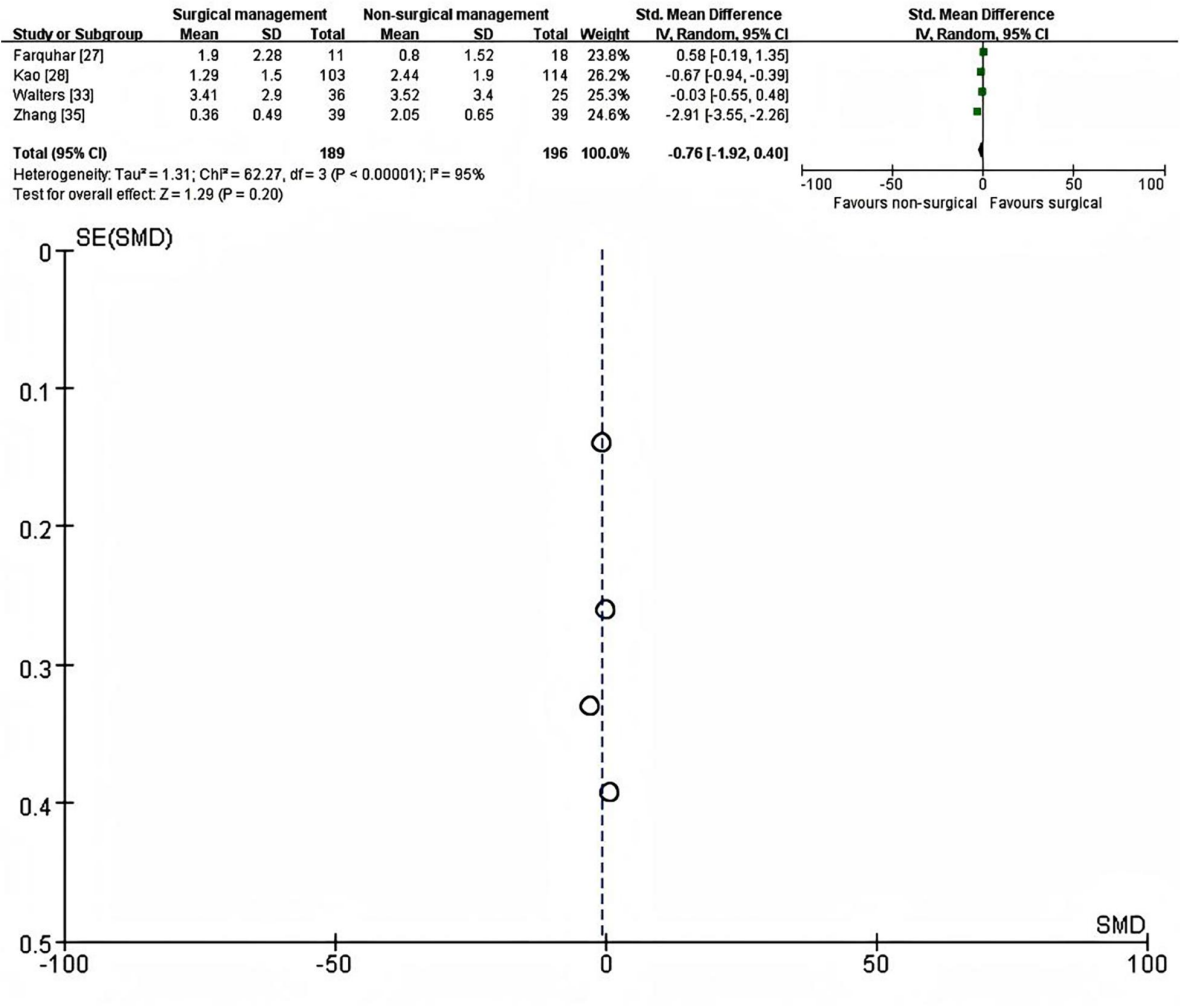
Forest plot (left) and galbraith plot (right) showing the random-effects meta-analysis of continuous chronic chest wall pain intensity scores assessed at >=3 months after SSRF versus non-operative management. SMD, standardized mean difference; CI, confidence interval; SE, standard error.

Because there was substantial heterogeneity between studies (I^2^ = 83%, *τ*^2^ = 0.30), a random-effects inverse-variance model with SMD as the effect measure was used. The pooled analysis demonstrated no statistically significant difference in chronic pain severity between SSRF and non-operative management at the longest follow-up (SMD = −0.11, 95% CI −0.80 to 0.58, *p* = 0.75) (Figure 4). A fixed-effect sensitivity analysis was performed as a robustness check. Under this model, the pooled estimate suggested lower chronic pain scores in the non-operative group (SMD = −0.42, 95% CI −0.66 to −0.19) (Supplementary Figure S5).

### Secondary outcome

#### Tracheostomy

**Figure 5:**
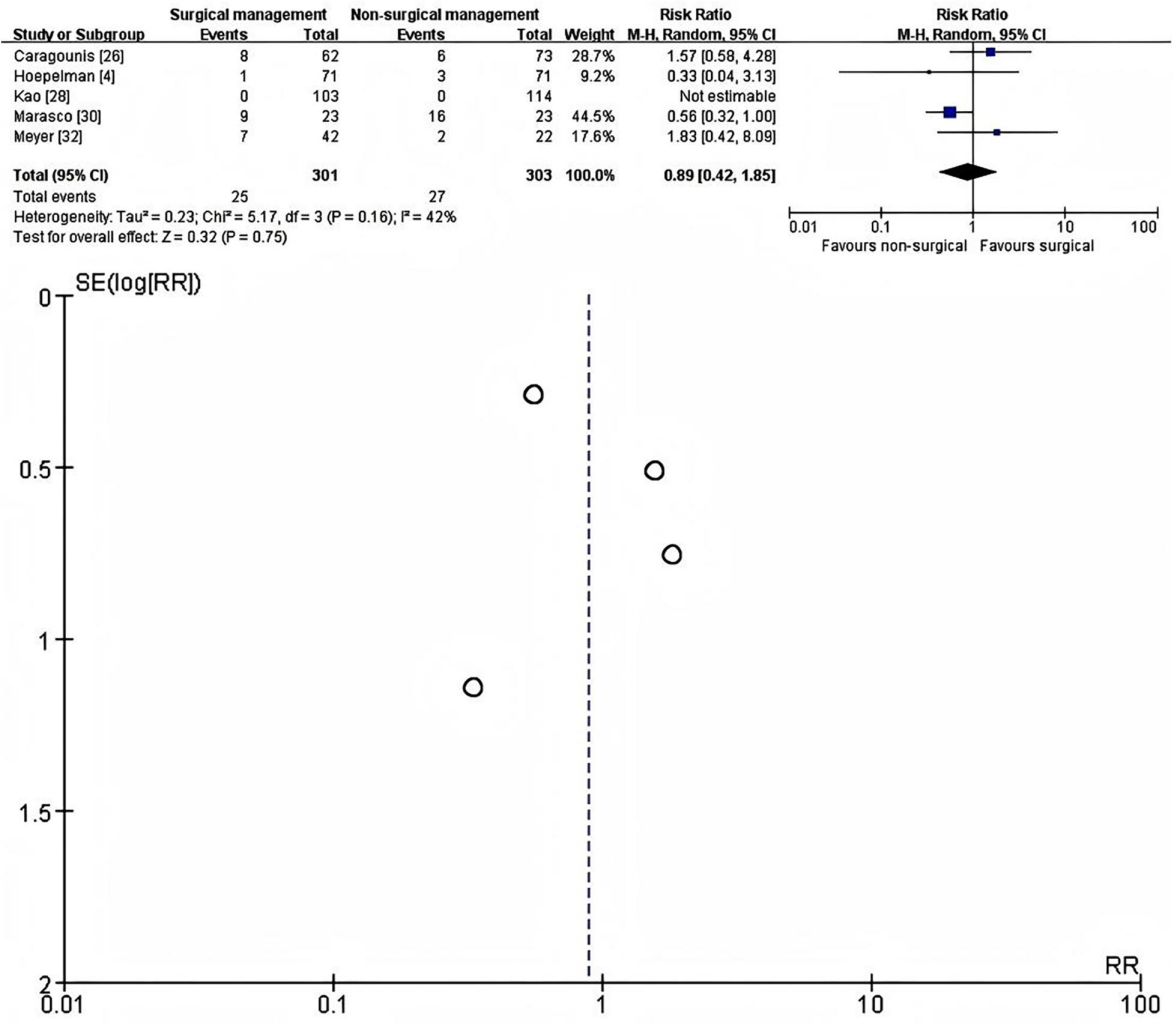
Forest plot (left) and galbraith plot (right) showing the random-effects meta-analysis of tracheostomy during the index admission after SSRF versus non-operative management. RR, risk ratio; CI, confidence interval; SE, standard error.

Five studies (4, 26, 28, 30, 32) reported the need for tracheostomy during the index hospital stay and were included in the quantitative analysis (301 patients treated with SSRF and 303 managed non-operatively). Tracheostomy was defined as the performance of a surgical airway during the acute admission, usually in the context of prolonged or anticipated prolonged mechanical ventilation. One study (28) reported zero tracheostomy events in both groups and therefore contributed to the total sample size but not to the pooled effect estimate. Using a random-effects model, there was no significant difference in tracheostomy rates between SSRF and non-operative management (RR = 0.89, 95% CI 0.42–1.85, *p* = 0.75; 25/301 vs. 27/303 events). Between-study heterogeneity was low-to-moderate (I^2^ = 42%, *τ*^2^ = 0.23, *χ*^2^ = 5.17, *p* = 0.16) (Figure 5). The overall number of tracheostomies was small, and the confidence interval was wide, indicating that current evidence does not demonstrate a clear reduction in the need for tracheostomy with SSRF compared with contemporary non-operative care.

## Discussion

In this systematic review and meta-analysis of 14 studies involving 1,947 adults with traumatic rib fractures, we evaluated the long-term patient-centred and clinical impact of SSRF compared with non-operative management. Overall, the available evidence does not demonstrate a clear, clinically meaningful benefit of SSRF for long-term HRQoL or chronic chest wall pain. After exclusion of one outlying cohort, pooled HRQoL estimates were neutral or slightly favoured non-operative care, with effect sizes that were small and unlikely to be perceptible in practice. Similarly, binary analyses suggested more frequent reports of chronic chest wall pain after SSRF, whereas continuous pain scores showed no consistent advantage of either strategy. Consistent with the study focus, we concentrated on long-term patient-reported outcomes and retained tracheostomy as the only secondary outcome with potential longer-term relevance. Viewed in context, these overarching patterns indicate that durable patient-reported benefits of SSRF remain uncertain and, if present, are likely modest; any potential gains should be weighed against operative risks and resource implications.

From a health-related quality of life perspective, our findings do not support the assumption that SSRF confers superior long-term patient-reported outcomes. In the primary analysis of seven comparative studies, standardized HRQoL scores assessed at 6–21 months were broadly similar between SSRF and non-operative management. Excluding a single outlying cohort shifted the pooled estimate slightly in favour of non-operative care, although the magnitude of this difference remained small and unlikely to be clinically meaningful. This pattern aligns with longer-term cohort evidence indicating that, while patients with multiple rib fractures may experience sustained impairment in generic HRQoL, operative fixation has not been consistently associated with higher SF-12 or EQ-5D scores compared with non-operative treatment. In Marasco's multicentre cohort of major trauma patients, SSRF was not associated with improved SF-12 or Glasgow Outcome Scale–Extended scores through 24 months of follow-up (16). Similarly, Prins et al. reported EQ-5D utilities and SF-12 component scores at a median follow-up of approximately 3 years, without a clear and consistent separation between operatively and non-operatively managed patients (6). Collectively, the available evidence suggests that any long-term HRQoL benefit attributable to SSRF—if present—is likely to be modest and should not be considered the primary rationale for operative stabilisation.

Substantial between-study heterogeneity was observed in the primary HRQoL meta-analysis (I^2^ = 89%). This likely reflects important clinical and methodological differences across studies, including variability in follow-up duration, the HRQoL instruments used, case-mix, and peri-injury analgesic protocols. In a prespecified subgroup restricted to studies that reported generic HRQoL measures at 6–12 months, statistical heterogeneity disappeared (I^2^ = 0%) and the pooled estimate remained imprecise without a consistent advantage for either strategy, whereas studies with longer follow-up suggested that any HRQoL benefit tends to diminish over time. This late convergence does not mean SSRF lacks clinical value in selected patients. The most plausible benefits occur early after injury, when chest wall stabilisation can improve respiratory mechanics and facilitate ventilator liberation in high-risk phenotypes (12). Early trials and meta-analyses suggest that such short-term physiological improvements may translate into meaningful gains during the acute recovery phase (9, 11). However, these early advantages may not persist as separations in generic HRQoL once fracture healing progresses and functional recovery trajectories converge over time. Other potential effect modifiers – such as patient age, injury severity scores, the proportion of flail chest, the timing of fixation (early ≤ 72 h vs. delayed), and the use of regional analgesia – also varied across cohorts but were reported too sparsely and inconsistently to allow informative meta-regression in this dataset. As a result, residual heterogeneity remains, and the pooled HRQoL estimates should be interpreted with caution. Importantly, any potential benefit is likely to occur early in recovery, when improved chest wall stability may facilitate ventilation, mobilisation, and rehabilitation, whereas very late follow-up may reflect convergence of recovery trajectories across treatment strategies. Notably, most studies did not provide sufficiently granular descriptions of chest wall injury severity or operative indications to permit subgroup analyses by flail chest status, rib-fracture burden/laterality, displacement severity, or global trauma burden. In addition, treatment decisions likely reflected heterogeneous local protocols and surgeon judgement rather than a uniform indication framework.

A key interpretive challenge is confounding by indication: patients undergoing SSRF often have more severe chest wall injury and greater overall trauma burden, and concomitant injuries may independently shape long-term HRQoL and pain. Because these factors were inconsistently reported and rarely adjusted for, the pooled comparisons should be interpreted as associations rather than causal effects.

Chronic chest wall pain was a prespecified primary outcome in the present review, and the pooled evidence suggests that SSRF does not reliably reduce—and may modestly increase—the long-term burden of pain. In the primary meta-analysis of binary outcomes at the longest available follow-up (≥3 months), approximately one third of survivors in each group reported persistent chest wall pain, with very similar crude proportions in the operative and non-operative arms (36% vs. 34%), yet the pooled risk ratio favoured non-operative management (RR ≈ 1.28, 95% CI 1.03–1.58) and statistical heterogeneity was negligible. Across individual studies, the prevalence of chronic pain at final assessment ranged from about 10% to 68% after SSRF and from 16% to 47% after non-operative care, reflecting substantial clinical heterogeneity. In Bauman's multicentre cohort, roughly one quarter of patients in both groups still reported chest wall pain 17–20 months after injury (22/78 vs. 48/174) (25), whereas both trials by Marasco and colleagues in flail chest and severe chest wall injury cohorts consistently showed higher rates of persistent pain in the surgically treated arms at 6–24 months (e.g., 9/16 vs. 191/445 at 24 months; 30/44 vs. 22/47 at 6 months) (30, 31). Continuous pain scores from four studies (27, 28, 33, 35) were highly variable and did not differ significantly between strategies in the random-effects model, despite some individual cohorts suggesting lower early mean pain scores after SSRF. These findings align with longer-term observational data showing that a substantial proportion of patients report residual thoracic pain several years after blunt chest trauma and that treatment modality is not a consistent determinant of late pain complaints (6).

Several mechanisms may account for the absence of a durable analgesic benefit with SSRF. Persistent pain after rib fractures is multifactorial and may be driven by residual chest wall deformity or instability, costovertebral joint involvement, intercostal nerve injury and pleural or myofascial scarring (15). Surgical exposure and plate fixation require additional soft-tissue dissection in close proximity to the intercostal neurovascular bundles, and long-term series of SSRF recipients have described non-trivial rates of implant-related discomfort, chest wall stiffness and late plate removal because of pain or foreign-body sensation (16, 26, 30, 31). In parallel, patients managed non-operatively often experience progressive fracture healing, compensatory musculoskeletal adaptation and functional recovery over time, potentially attenuating any early differences in pain trajectories between strategies. On this basis, SSRF should not be offered with the expectation of preventing chronic chest wall pain; instead, patients—particularly those with flail segments or markedly displaced fractures—should be counselled that long-term chest wall symptoms remain common irrespective of treatment approach and that structured, multimodal pain management is likely to be required whether or not operative stabilisation is undertaken. In addition, baseline injury severity is a major determinant of long-term pain: patients selected for SSRF often have more extensive displacement and soft-tissue or intercostal nerve injury, which may predispose to chronic neuropathic pain regardless of fixation. The limited granularity of pain reporting in the available literature, particularly the sparse use of standardized intensity thresholds and neuropathic pain instruments, restricts inference about clinically meaningful differences in pain burden.

Over the past decade, these trial and meta-analytic data have been incorporated into evolving chest-trauma pathways and guideline recommendations, which generally advise that SSRF should be considered in patients with ventilator-dependent flail chest or severe rib displacement when appropriate surgical expertise and institutional infrastructure are available (3, 36, 37, 39, 40). In parallel, non-operative management of rib fractures has improved substantially, with guideline-driven chest-trauma protocols emphasising protocolised multimodal analgesia, regional anaesthetic techniques, early mobilisation and respiratory physiotherapy; recent systematic reviews of non-pharmacological interventions highlight that optimised conservative care alone can improve pain control, functional recovery and complication rates in many patients (37–39). Against this backdrop of enhanced surgical and non-surgical strategies, it is no longer self-evident that adding SSRF will translate into superior long-term patient-reported outcomes across the broader traumatic rib-fracture population.

Our study adds to the existing literature by systematically synthesising all available data on long-term HRQoL and chronic chest wall pain after SSRF vs. non-operative care—outcomes that have been largely under-reported in prior trials and meta-analyses focused on short-term physiological endpoints (36, 39, 40). The present findings suggest that, even if SSRF offers short-term benefits in carefully selected flail-chest populations, these advantages do not automatically translate into durable improvements in generic HRQoL or chronic pain when compared with contemporary guideline-driven non-operative management. SSRF should therefore not be portrayed as a universally superior standard of care for all patients with multiple rib fractures, but rather as a potentially useful intervention for selected high-risk patients, with the important caveat that its long-term patient-centred benefits appear limited and, in the case of chronic pain, may even be unfavourable in some subgroups. In this context, high-risk phenotypes refer to severe, clearly characterized chest wall injuries for which operative stabilisation is generally considered to provide short-term physiological benefit, such as ventilator-dependent flail chest, an unstable chest wall with paradoxical motion, or markedly displaced fractures causing refractory respiratory compromise, rather than routine fixation of all rib fractures.

From a clinical decision-making perspective, our results support a more selective and nuanced use of SSRF. Existing guidelines and expert reviews endorse surgery as a valuable option in clearly defined high-risk phenotypes—such as ventilator-dependent flail chest, unstable chest wall injuries with paradoxical motion, or markedly displaced fractures causing refractory respiratory compromise—where short-term improvements in respiratory mechanics are most likely to yield tangible benefit (1–3, 8, 12, 16, 37, 40–43). In contrast, for the much larger group of patients with intermediate-severity injuries who maintain adequate gas exchange and pain control under optimised non-operative protocols, the incremental benefit of SSRF is uncertain and may not justify the additional operative risk and resource utilisation. These considerations are directly relevant to preoperative counselling, shared decision-making and service planning: clinicians should communicate that long-term HRQoL is unlikely to be improved by SSRF, that chronic chest wall pain remains common regardless of treatment, and that SSRF is a resource-intensive intervention whose indications should be refined according to local critical care capacity, patient frailty, fracture pattern and overall injury burden rather than applied as a one-size-fits-all solution.

Beyond the clinical insights, this study has several notable strengths. First, to our knowledge it is the first systematic review and meta-analysis explicitly designed around long-term HRQoL and chronic chest wall pain after SSRF vs. non-operative management, rather than short-term physiological endpoints alone. The protocol was prospectively registered, the review adhered to PRISMA 2020 guidance, and a comprehensive multi-database search was performed with independent, duplicate screening, data extraction and quality assessment, which strengthens the internal validity and reproducibility of the findings. Second, we prespecified HRQoL and chronic pain as primary outcomes, used the longest available follow-up (≥3 months), and synthesised binary and continuous pain measures separately with sensitivity analyses, allowing a more nuanced appraisal of long-term patient-reported outcomes. Finally, we retained tracheostomy as a secondary outcome to capture prolonged ventilatory dependence, while the main analyses focus on long-term patient-reported outcomes.

Several limitations should be acknowledged. Most included studies were observational cohorts, making the results vulnerable to selection bias and residual confounding by indication, as patients selected for SSRF typically had more severe chest wall injury and higher baseline risk than those treated non-operatively. There was substantial clinical and methodological heterogeneity in inclusion criteria, fracture patterns, timing and technique of fixation, analgesic and rehabilitation protocols, and in the instruments and thresholds used to measure HRQoL and chronic pain, which may have diluted modest but clinically relevant effects. For tracheostomy, event numbers were small and sample sizes limited, resulting in wide confidence intervals and low certainty in the pooled estimate. Finally, follow-up durations varied and truly long-term outcomes beyond two years were rarely reported, so the evolution of HRQoL, pain and implant-related symptoms over very late time horizons remains uncertain. Critically, key descriptors of chest wall injury severity and overall trauma burden were inconsistently reported, limiting assessment of baseline comparability and effect modification. These include the presence of flail chest, the number and laterality of fractured ribs, the degree of displacement, and validated trauma metrics such as chest Abbreviated Injury Scale and Injury Severity Score; rib-specific composite scores were rarely available. Pain outcomes were also variably defined: binary “pain present/absent” measures may conflate mild and severe symptoms, and continuous intensity scores were reported on heterogeneous scales with limited detail on neuropathic features or analgesic co-interventions. Finally, technical details of osteosynthesis were incompletely reported, including the implant system, surgical approach, number of ribs fixed, fixation strategy, and late hardware-related symptoms or removals, limiting assessment of procedure-level effect modification.

In conclusion, based on the available comparative data, SSRF has not been shown to confer consistent, clinically important improvements in long-term HRQoL or chronic chest wall pain beyond 3 months compared with contemporary non-operative management; however, inference is constrained by heterogeneity and confounding by injury severity and indication. The finding that a modestly higher proportion of patients report persistent chest wall pain after SSRF suggests that routine expansion of SSRF to unselected rib-fracture populations is not justified. Instead, the role of SSRF should remain focused on carefully defined high-risk phenotypes, and future research should prioritise well-designed prospective studies and adequately powered randomised trials incorporating standardized long-term patient-reported outcomes and formal cost-effectiveness analyses to clarify in which subgroups, if any, SSRF provides durable, patient-centred benefit. These findings should be interpreted as pertaining to long-term patient-reported outcomes and do not negate established short-term physiological benefits of SSRF in selected severe injuries; rather, they underscore the need to define whether early gains translate into durable, patient-centred benefit across clearly characterised injury phenotypes.

## Data Availability

The original contributions presented in the study are included in the article/Supplementary Material, further inquiries can be directed to the corresponding author.

## Supplementary material

Supplementary Figure S1 Sensitivity analysis of HRQoL comparing SSRF with non-operative management using a fixed-effect model, shown as a forest plot (left) with the corresponding Galbraith plot (right). HRQoL, health-related quality of life; SSRF, surgical stabilisation of rib fractures; SMD, standardized mean difference; CI, confidence interval; SE, standard error.

Supplementary Figure S2 Sensitivity analysis of HRQoL after SSRF vs. non-operative management restricted to four comparative studies, using a random-effects model and shown as a forest plot (left) with the corresponding Galbraith plot (right). HRQoL, health-related quality of life; SSRF, surgical stabilisation of rib fractures; SMD, standardized mean difference; CI, confidence interval; SE, standard error.

Supplementary Figure S3 Sensitivity analysis of HRQoL in the same four studies using a fixed-effect model, shown as a forest plot (left) with the corresponding Galbraith plot (right). HRQoL, health-related quality of life; SSRF, surgical stabilisation of rib fractures; SMD, standardized mean difference; CI, confidence interval; SE, standard error.

Supplementary Figure S4 Sensitivity analysis of HRQoL restricted to studies with follow-up ≥12 months, comparing SSRF with non-operative management using a fixed-effect model, shown as a forest plot (left) with the corresponding Galbraith plot (right). HRQoL, health-related quality of life; SSRF, surgical stabilisation of rib fractures; SMD, standardized mean difference; CI, confidence interval; SE, standard error.

Supplementary Figure S5 Sensitivity analysis of continuous chronic chest wall pain intensity scores at ≥3 months after SSRF vs. non-operative management, shown as a random-effects forest plot (left) with the corresponding Galbraith plot (right). HRQoL, health-related quality of life; SSRF, surgical stabilisation of rib fractures; SMD, standardized mean difference; CI, confidence interval; SE, standard error.
