# Supplementary material for: Long-term quality of life and chronic pain after surgical vs. non-operative treatment of rib fractures: systematic review and meta-analysis
Source: Front Surg. 2026 Mar 30;13:1774082. doi: 10.3389/fsurg.2026.1774082 (PMC13070923; doi:10.3389/fsurg.2026.1774082)
Supplement: Supplementary Figure S1 — Sensitivity analysis of HRQoL comparing SSRF with non-operative management using a fixed-effect model, shown as a forest plot (left) with the corresponding Galbraith plot (right). HRQoL, health-related quality of life; SSRF, surgical stabilisation of rib fractures; SMD, standardized mean difference; CI, confidence interval; SE, standard error. [file Supplementaryfile1.zip › Supplementary file 1.docx]

### Supplementary Methods: Literature search strategy

We systematically searched PubMed, Embase, Scopus, Web of Science Core Collection and the Cochrane Library to identify studies evaluating long-term health-related quality of life or chronic pain after surgical versus non-operative management of traumatic rib fractures. No language restrictions other than English were applied. The searches were last updated on **31 October 2025**. The detailed search strategies for each database are provided below.

#### 1. Cochrane Library

**Database:** Cochrane Library (CENTRAL and other Cochrane databases)
**Search date:** up to 31 October 2025

**Step 1 – Traumatic rib fractures (#1)**

"rib fracture*" OR "flail chest" OR "chest wall fracture*" OR "rib injur*" OR "rib trauma"

**Step 2 – Surgical stabilization / fixation (#2)**

"surgical stabilization of rib fracture*" OR "surgical stabilisation of rib fracture*" OR "surgical rib fixation" OR "rib fixation" OR "rib plating" OR "rib osteosynthesis"
OR "rib stabilization" OR "rib stabilisation"

**Step 3 – HRQoL and chronic pain (#3)**

"quality of life" OR "health-related quality of life" OR HRQoL OR "EQ-5D" OR "EQ 5D" OR "SF-36" OR "SF 36" OR "SF-12" OR "SF 12" OR PROMIS OR "patient-reported outcome*" OR "patient reported outcome*" OR "functional outcome*" OR "long-term outcome*" OR "long term outcome*" OR "long-term follow-up" OR "long term follow up" OR "chronic pain" OR "persistent pain" OR "chest wall pain" OR "rib pain"

**Final combination**

#1 AND #2 AND #3

#### 2. Embase

**Database:** Embase (via Ovid or Elsevier)
**Search date:** up to 31 October 2025

**Step 1 – Traumatic rib fractures (#1)**

('rib fracture'/exp OR 'thoracic wall injury'/exp OR 'flail chest'/de OR 'rib fracture*' OR 'flail chest' OR 'chest wall fracture*' OR 'rib injur*' OR 'rib trauma' OR ('multiple' AND 'rib fracture*'))

**Step 2 – Surgical stabilization / fixation (#2)**

('fracture fixation'/exp OR 'internal fixation'/exp OR 'bone plating'/exp OR 'thoracic wall surgery'/exp OR 'rib fracture fixation'/de OR 'surgical stabilization of rib fracture*' OR 'surgical stabilisation of rib fracture*' OR 'surgical rib fixation' OR 'rib fixation' OR 'rib plating' OR 'rib osteosynthesis' OR 'rib stabilization' OR 'rib stabilisation' OR ((surgery OR surgical OR operative) AND (fixation OR stabilization OR stabilisation OR plating OR osteosynthesis) AND (rib OR ribs OR 'chest wall')))

**Step 3 – HRQoL and chronic pain (#3)**

('quality of life'/exp OR 'chronic pain'/exp OR 'patient reported outcome'/exp
OR 'quality of life' OR 'health-related quality of life' OR hrqol OR 'EQ-5D' OR 'EQ 5D' OR 'SF-36' OR 'SF 36' OR 'SF-12' OR 'SF 12' OR promis OR 'patient-reported outcome*' OR 'patient reported outcome*' OR 'functional outcome*' OR 'long-term outcome*' OR 'long term outcome*' OR 'long-term follow-up' OR 'long term follow up' OR 'chronic pain' OR 'persistent pain' OR 'chest wall pain' OR 'rib pain')

**Final combination**

#1 AND #2 AND #3

#### 3. PubMed

**Database:** PubMed (MEDLINE)
**Date range:** from database inception to 31 October 2025
**Search fields:** MeSH terms and Title/Abstract

**Step 1 – Traumatic rib fractures (#1)**

"Rib Fractures"[Mesh] OR rib fracture*[tiab] OR rib injur*[tiab] OR rib trauma[tiab]
OR "chest wall fracture*"[tiab] OR "flail chest"[tiab]

**Step 2 – Surgical stabilization / fixation (#2)**

"Fracture Fixation"[Mesh] OR "Thoracic Wall/surgery"[Mesh] OR "Orthopedic Procedures"[Mesh] OR (surgical[tiab] AND (stabilization[tiab] OR stabilisation[tiab] OR fixation[tiab])) OR "surgical stabilization of rib fracture*"[tiab] OR "surgical stabilisation of rib fracture*"[tiab] OR "surgical rib fixation"[tiab] OR "rib fixation"[tiab] OR "rib plating"[tiab] OR "rib osteosynthesis"[tiab] OR "rib stabilization"[tiab]

**Step 3 – HRQoL and chronic pain (#3)**

"Quality of Life"[Mesh] OR "Health Status"[Mesh] OR "Patient Reported Outcome Measures"[Mesh] OR "Chronic Pain"[Mesh] OR "Pain, Postoperative"[Mesh] OR "quality of life"[tiab] OR "health-related quality of life"[tiab] OR HRQoL[tiab] OR "EQ-5D"[tiab] OR "EQ 5D"[tiab] OR "SF-36"[tiab] OR "SF 36"[tiab] OR "SF-12"[tiab] OR "SF 12"[tiab] OR PROMIS[tiab] OR "patient-reported outcome*"[tiab] OR "patient reported outcome*"[tiab] OR "functional outcome*"[tiab] OR "long-term outcome*"[tiab] OR "long term outcome*"[tiab] OR "long-term follow-up"[tiab] OR "long term follow up"[tiab] OR "chronic pain"[tiab]
OR "persistent pain"[tiab] OR "chest wall pain"[tiab] OR "rib pain"[tiab]

**Final combination**

#1 AND #2 AND #3

#### 4. Scopus

**Database:** Scopus
**Search date:** up to 31 October 2025

**Block 1 – Traumatic rib fractures (S1)**

TITLE-ABS-KEY("rib fracture*" OR "rib injur*" OR "rib trauma" OR "chest wall fracture*" OR "flail chest" OR ("multiple" W/3 "rib fracture*"))

**Block 2 – Surgical stabilization / fixation (S2)**

TITLE-ABS-KEY("surgical stabilization of rib fracture*" OR "surgical stabilisation of rib fracture*" OR "surgical rib fixation" OR "rib fixation" OR "rib plating" OR "rib osteosynthesis" OR "rib stabilization" OR "rib stabilisation" OR ( (surgery OR surgical OR operative) W/3 (stabilization OR stabilisation OR fixation OR plating OR osteosynthesis) AND (rib OR ribs OR "chest wall") ) )

**Block 3 – HRQoL and chronic pain (S3)**

TITLE-ABS-KEY("quality of life" OR "health-related quality of life" OR HRQoL OR "EQ-5D" OR "EQ 5D" OR "SF-36" OR "SF 36" OR "SF-12" OR "SF 12" OR PROMIS OR "patient-reported outcome*" OR "patient reported outcome*" OR "functional outcome*" OR "long-term outcome*" OR "long term outcome*" OR "long-term follow-up" OR "long term follow up" OR "chronic pain" OR "persistent pain" OR "chest wall pain" OR "rib pain" )

**Final combination**

S4 = S1 AND S2 AND S3

#### 5. Web of Science Core Collection

**Database:** Web of Science Core Collection
**Search field:** Topic (ALL)
**Search date:** up to 31 October 2025

**Step 1 – Traumatic rib fractures (S1)**

ALL = ("rib fracture*" OR "rib injur*" OR "rib trauma" OR "chest wall fracture*" OR "flail chest" OR ("multiple" AND "rib fracture*") )

**Step 2 – Surgical stabilization / fixation (S2)**

ALL = ("surgical stabilization of rib fracture*" OR "surgical stabilisation of rib fracture*" OR "surgical rib fixation" OR "rib fixation" OR "rib plating" OR "rib osteosynthesis" OR "rib stabilization" OR "rib stabilisation" OR ( (surgery OR surgical OR operative) AND (stabilization OR stabilisation OR fixation OR plating OR osteosynthesis) AND (rib OR ribs OR "chest wall") ) )

**Step 3 – HRQoL and chronic pain (S3)**

ALL = ("quality of life" OR "health-related quality of life" OR HRQoL OR "EQ-5D" OR "EQ 5D" OR "SF-36" OR "SF 36" OR "SF-12" OR "SF 12" OR PROMIS OR "patient-reported outcome*" OR "patient reported outcome*" OR "functional outcome*" OR "long-term outcome*" OR "long term outcome*" OR "long-term follow-up" OR "long term follow up" OR "chronic pain" OR "persistent pain" OR
"chest wall pain" OR "rib pain" )

**Final combination**

S1 AND S2 AND S3
