# Supplementary material for: Long-term quality of life and chronic pain after surgical vs. non-operative treatment of rib fractures: systematic review and meta-analysis
Source: Front Surg. 2026 Mar 30;13:1774082. doi: 10.3389/fsurg.2026.1774082 (PMC13070923; doi:10.3389/fsurg.2026.1774082)
Supplement: Supplementary Figure S1 — Sensitivity analysis of HRQoL comparing SSRF with non-operative management using a fixed-effect model, shown as a forest plot (left) with the corresponding Galbraith plot (right). HRQoL, health-related quality of life; SSRF, surgical stabilisation of rib fractures; SMD, standardized mean difference; CI, confidence interval; SE, standard error. [file Supplementaryfile1.zip › Table 1.docx]

| Supplementary Table S1. Detailed treatment characteristics and outcome data of included studies comparing SSRF with non-operative management in adults with traumatic rib fractures | | | | | | | | | | | | | | | | | | | | |
| --- | --- | --- | --- | --- | --- | --- | --- | --- | --- | --- | --- | --- | --- | --- | --- | --- | --- | --- | --- | --- |
| Study Name | Year | Treatment Type | Surgical Technique | Medication Used | HRQoL Score (3 months) | Pain Score (NRS) (3 months) | HRQoL Score (6 months) | Pain Score (NRS) (6 months) | HRQoL Score (12 months) | Pain Score (NRS) (12 months) | Long-term Pain Score | Hospitalization Days | ICU Stay Days | Mechanical Ventilation | Pneumonia Rate | Mortality Rate | Chronic Pain (%) | Functional Recovery | Complication Rate | Chest Wall Deformity |
| Bauman et al [25] | 2022 | Surgical stabilization of rib fractures (SSRF) vs Non-operative (NO) | Muscle-sparing SSRF | Painkillers, Gabapentin, NSAIDs, Muscle Relaxants, Opioids, Epidural | Not provided | Not provided | Not provided | Not provided | Not provided | Not provided | 2 (1–4) SSRF vs 4 (2–6) Non-operativeve | 7.83 (7.91) SSRF vs 10.19 (7.60) Non-operative | 3.13 (6.00) SSRF vs 2.13 (4.27) Non-operative | 1.68 (4.87) SSRF vs 1.25 (4.58) Non-operative | Not provided | Not provided | 28.2% SSRF vs 27.6% Non-operative | 75.6% SSRF vs 56.3% Non-operative | Not provided | 13.8% Non-operative vs 3.8% SSRF |
| Caragounis et al [26] | 2025 | Operative (SSRF) vs Non-operative management of flail chest | Plate fixation with MatrixRIB system; mainly thoracotomy early, later muscle-sparing without thoracotomy | Paracetamol, NSAIDs, opioids, thoracic epidural analgesia; some Operative patients with intrapleural local anaesthetic | Not provided | Not provided | 0.76 (0.12) Operative vs 0.76 (0.13) Non-operative(EQ-5D-5L Index, 6 months) | Not provided | 0.78 (0.14) Operative vs 0.77 (0.15) Non-operative(EQ-5D-5L Index, 12 months) | Not provided | Not provided | Median 14 (7.2–44.9) Operative vs 11 (2.7–41.6) Non-operative | Median 6.0 (1.0–40.8) Operative vs 5.0 (1.0–31.2) Non-operative | 29.0% Operative vs 29.2% Non-operative need MV; MV days 8.5 (1.0–40.5) Operative vs 8.0 (1.0–31.3) Non-operative | 11/62 (17.7%) Operative vs 27/73 (37.0%) Non-op | 30-d 3.2% Operative vs 2.7% Non-operative; 1-yr 8.1% Operative vs 6.8% Non-operative | 10% Operative vs 16% Non-operative (pain when breathing at 1 year) | Working at 1 year: 89% Operative vs 100% Non-operative | Not provided | Not provided |
| Farquhar et al [27] | 2016 | Surgical fixation with MatrixRIB plates vs nonoperative comprehensive management | Open fixation of ≥3 fractured ribs using Synthes MatrixRIB locking plates | Multimodal analgesia per EAST guidelines, including epidural analgesia, systemic opioids and chest physiotherapy (exact drug doses not specified) | Not provided | Not provided | Not provided | Not provided | Not provided (long-term EQ-5D-5L domain scores reported without a precise time point) | Not provided | VAS chest pain 1.9 (cases) vs 0.8 (controls) | 21.9 ± 13.2 days Operative vs 16.0 ± 12.1 days Non-operative | 7.4 ± 6.7 days Operative vs 3.7 ± 6.0 days Non-operative | 79% Operative vs 36% Nonoperative eventually required intubation; ventilator days 6.1 ± 5.9 vs 3.1 ± 5.5 | 12/19 (63%) Operative vs 8/36 (22%) Non-operative | 1/19 (5%) Operative vs 1/36 (4%) Non-operative | Not provided as a percentage; long-term VAS chest pain 1.9 Operative vs 0.8 Nonoperative | Return to employment: 36% Operative vs 23% Non-operative | Not provided | Not provided |
| Hoepelman et al [4] | 2023 | Rib fixation (SSRF) vs Non-operative treatment for CT-confirmed multiple rib fractures without clinical flail chest | Open rib fixation using plate osteosynthesis according to a standardized algorithm (locking plates such as MatrixRIB; fixation of selected fractured ribs) | Non-operative treatment consisted of adequate multimodal pain management (epidural and intravenous analgesia), supportive mechanical ventilation when needed, and physiotherapy for breathing exercises according to national guidelines | Not provided (EQ-5D-5L measured at 6 weeks and 12 months only) | Not provided | Not provided (6-week and 12-month EQ-5D-5L only; no 6-month time point) | Not provided | EQ-5D-5L index value 0.81 ± 0.2 Non-operative vs 0.74 ± 0.2 Rib fixation at 1 year | NRS general pain 0 (IQR 0–2) Non-operative vs 2 (IQR 0–4) Rib fixation at 1 year | At 1 year: NRS general pain 0 (0–2) Non-operative vs 2 (0–4) Rib fixation; NRS pain with coughing 0 (0–0) Non-operative vs 0 (0–1) Rib fixation | Hospital length of stay: median 9 days (IQR 6–13) Non-operative vs 12 days (IQR 8–18) Rib fixation; regression coefficient +4.9 days (95% CI 0.8–9.1, p = 0.019) in favor of non-operative treatment | ICU length of stay: median 2 days (IQR 1–6) Non-operative vs 4 days (IQR 2–11) Rib fixation (after propensity score matching) | Duration of invasive mechanical ventilation: median 4 days (IQR 2–5) Non-operative vs 6 days (IQR 3–12.5) Rib fixation; proportion of patients requiring invasive ventilation not explicitly reported for the full matched cohort (only 15.8% vs 22.0% in the ≤72 h surgery subgroup) | In-hospital pneumonia 12.6% (approx. 17.1%) Non-operative vs 21.4% (approx. 30.2%) Rib fixation (difference not statistically significant; OR 2.1, 95% CI 0.8–5.6, p = 0.123) | In-hospital mortality 1.6% (approx. 2.3%) Non-operative vs 1.9% (approx. 2.7%) Rib fixation; at 1-year follow-up: 1.3% Non-operative vs 0.8% Rib fixation | Not provided as a percentage; only NRS scores at 6 weeks and 12 months (see Long-term Pain Score) | Return to work: median 12 weeks (IQR 7–20) Non-operative vs 12 weeks (IQR 10–20) Rib fixation; Return to sports: median 14 weeks (IQR 8–26) Non-operative vs 12 weeks (IQR 10–20) Rib fixation | In-hospital “other complications” 20.3 events (28.6%) Non-operative vs 31.4 events (43.7%) Rib fixation; overall composite complication rate not reported as a single summarized percentage | Symptomatic non-union at 1 year 1.2% Non-operative vs 1.0–1.4% Rib fixation; explicit chest wall deformity (cosmetic) not reported |
| Kao et al [28] | 2025 | Surgical stabilization of rib fractures (SSRF) vs conservative treatment | Open SSRF with metallic plates and screws plus video-assisted thoracoscopy as needed | Multimodal analgesia and supportive care (details of specific drugs not clearly reported) | Not provided | 1.29 ± 1.50 Operative vs 2.44 ± 1.90 Non-operative | Not provided | Not provided | Not provided | Not provided | NRS at 3 months: 1.29 ± 1.50 Operative vs 2.44 ± 1.90 Non-operative | 12.97 ± 8.04 days Operative vs 7.99 ± 8.64 days Non-operative | 4.35 ± 4.76 days Operative vs 1.52 ± 3.06 days Non-operative | Intubation 35/103 (34.0%) Operative vs 9/114 (7.9%) Non-operative; duration of ventilation not reported | Postoperative pulmonary complications (pneumonia or hemothorax) 2/103 (1.9%) in operative group; not reported for non-operative group | Not provided | Not provided | Not provided | Postoperative complications 2/103 (1.9%) in operative group (subcutaneous hematoma / pulmonary complications); complications for non-operative group not reported | Not provided |
| Lian et al [29] | 2023 | Surgical rib fixation vs nonoperative treatment for moderately severe traumatic rib fractures | Open rib fixation; video-assisted thoracoscopic localization and evacuation of hemothorax; plates (SternaLock or MatrixRIB) fixed across fractures with ≥3 screws each side | Both groups received tube thoracostomy, intravenous and oral analgesia, external thoracic banding, and aggressive respiratory care | Not provided | Not provided | Not provided | Not provided | Not provided | Not provided | Not provided | Mean 14.97 days Operative vs 7.76 days Non-operative (SD not reported) | Mean 5.18 days Operative vs 1.45 days Non-operative (SD not reported) | Mean intubation time 49.1 hours Operative vs 2.95 hours Nonoperative (proportion requiring intubation not clearly reported) | 1/34 (2.9%) Operative vs 0/38 (0%) Non-operative at baseline/admission (no separate postoperative pneumonia incidence reported) | Not provided | Not provided | Not provided | Not provided | Not provided |
| Marasco et al [16] | 2013 | Operative rib fixation vs nonoperative best-practice mechanical ventilator management for flail chest | Open fixation of ribs 3–10 using Inion OTPS resorbable 6- or 8-hole plates and bicortical screws; usually 4 ribs fixed (range 2–7), mainly anterior/lateral fractures | Both groups received protocolized ICU care with sedation, analgesia and ventilator weaning; specific analgesic drugs not explicitly detailed | Not provided | Not provided | SF-36 at 6 months: PCS 33.6 ± 9.8 Operative vs 35.2 ± 10.7 Non-operative; MCS 45.1 ± 13.8 Operative vs 45.2 ± 9.2 Nonoperative | Not provided | Not provided | Not provided | SF-36 bodily pain domain at 6 months: 42.2 ± 9.4 Operative vs 37.9 ± 11.0 Non-operative (higher score = less pain) | Duration of hospital stay: median 20 days (IQR 18–28) Operative vs 25 days (IQR 18–38) Non-operative | Total ICU stay: median 324 h (IQR 238–380) Operative vs 448 h (IQR 323–647) Non-operative ≈ 13.5 vs 18.7 days | Duration of invasive mechanical ventilation postrandomization 151.8 ± 83.1 h Operative vs 181.0 ± 130.2 h Non-operative; noninvasive ventilation after extubation 57% vs 83%, median 3 h (0–25) vs 50 h (17–102); tracheostomy 9/23 (39%) vs 16/23 (70%) | 11/23 (48%) Operative vs 17/23 (74%) Non-operative | In-hospital mortality 0/23 (0%) Operative vs 1/23 (4.3%) Non-operative | Not provided as a percentage; authors state that most ongoing limitations at 3 months were due to other injuries rather than rib pain | Ongoing limitation in daily work/home life at 3 months: 10/21 (48%) Operative vs 15/21 (71%) Nonoperativ | No single overall rate reported; key complications include pneumonia 48% vs 74%, failed extubation 13% vs 4%, tracheostomy 39% vs 70%, readmission to ICU 9% vs 9% | Visible flail segment at 3 months: 0/21 Operative vs 2/21 Nonoperative; cosmetic chest appearance concerns 1 patient in each group |
| Marasco et al [30] | 2018 | Surgical rib fixation vs no rib fixation in major trauma patients with multiple rib fractures (thoracic AIS ≥3) | Operative rib fixation (plates) in ventilated flail chest or selected painful/displaced fractures; exact implant type and number of ribs fixed not specified | Both groups received specialist pain-team multimodal analgesia (oral and IV opioids including PCA, ketamine infusions, GABA analogues, NSAIDs, epidural or paravertebral local anaesthetic) plus chest physiotherapy:contentReference[oaicite:4]{index=4} | Not provided | Not provided | Not separately provided; SF-12 PCS and MCS reported as longitudinal least-squares means over 6–24 months (PCS 41.1 vs 38.7; MCS 51.5 vs 52.0, No fixation vs fixation):contentReference[oaicite:5]{index=5} | Not separately provided; mean NRS pain over 6–24 months 2.3 (2.0–2.7) No fixation vs 2.2 (1.4–2.9) Rib fixation (multivariable model) | Not provided | Not provided | Longitudinal NRS pain (0–10) mean 2.3 (95% CI 2.0–2.7) No fixation vs 2.2 (1.4–2.9) Rib fixation over 6–24 months | Not provided | ICU stay 173 [103–309] hours No fixation vs 251 [183–423] hours Rib fixation (medians; ≈7.2 vs 10.5 days) | Invasive mechanical ventilation 23.5% (333/1415) No fixation vs 61.2% (41/67) Rib fixation; MV duration 89 [33–187] hours No fixation vs 157 [98–309] hours Rib fixation (medians) | Not provided | 2.1% (30/1415) No fixation vs 1.5% (1/67) Rib fixation (in-hospital mortality) | Any pain at 24 months: 42.9% (191/445) No fixation vs 56.3% (9/16) Rib fixation; at 12 months: 43.1% (292/677) No fixation vs 42.1% (16/38) Rib fixation | GOSE 7–8 (good recovery) at 24 months: 38.1% (304/798) No fixation vs 33.3% (10/30) Rib fixation; return to work among those working pre-injury: 58% No fixation vs 47% Rib fixation at 12 months | Not provided | Not provided |
| Marasco et al [31] | 2022 | Rib fixation (SSRF) vs non-operative management in non-ventilator dependent multiple rib fractures | Surgical rib fixation with plated devices (RibLoc, MatrixRib, RibFix Blu, StraCos); median 3 [3–4] ribs fixed, mainly lateral then anterior | Protocolised multimodal analgesia with oral/IV opioids and optional epidural or paravertebral regional anaesthesia | SF-12 PCS 42.9 (10.0) Operative vs 44.4 (9.6) Non-operative; MCS 51.8 (9.5) Operative vs 50.9 (10.0) Non-operative | McGill Pain Rating Index median 7.7 [1.7–19.8] Operative vs 3.6 [0.7–15.3] Non-op erative | SF-12 PCS 44.4 (11.5) Operative vs 47.6 (9.5) Non-operative; MCS 51.6 (10.1) Operative vs 50.5 (11.9) Non-operative | McGill Pain Rating Index median 2.6 [0–17.1] Operative vs 0 [0–5.7] Non-operative | Not provided | Not provided | Prolonged pain (PRI > 8): 22/61 (36.1%) Operative vs 19/63 (30.2%) Non-operative at 3 months; 17/61 (27.9%) vs 9/63 (14.3%) at 6 months | Hospital LOS median 10 [7–13.5] days Operative vs 9 [6–13] days Non-operative | ICU LOS median 1 [0–2] days Operative vs 0 [0–2] days Non-operative | Not provided (non-ventilator dependent at enrolment; subsequent MV not reported by group) | 3 cases of pneumonia reported in cohort; group-specific rates not provided | In-hospital mortality 0/61 (0%) Operative vs 2/63 (3.3%) Non-operative | PRI > 8 at 6 months: 27.9% (17/61) Operative vs 14.3% (9/63) Non-operative | Return to work: 48.7% (19/39) Operative vs 40% (18/45) Non-operative at 3 months; 65.7% (23/35) vs 36.8% (14/38) at 6 months | Whole cohort complications: wound infection (1), pneumonia (3), bacteraemia (1), failed extubation requiring re-intubation (2), postoperative haemothorax (1); rates by group not clearly reported | Not provided |
| Meyer et al [32] | 2023 | SSRF vs usual care (Non-operative management) for severe chest wall injury without clinical flail chest | Surgical stabilization of at least one true rib using any commercial rib fixation system; muscle-sparing, small incisions | Protocolized multimodal pill-based analgesia (acetaminophen, NSAID, GABA analog, lidocaine patch; opioids, IV lidocaine/ketamine or regional blocks as needed) | EQ-5D-5L Index 0.61 (0.36) Usual care vs 0.56 (0.32) SSRF; VAS 73 (23) vs 69 (24) | Not provided (only EQ-5D-5L pain dimension 2 (2–3) Usual care vs 3 (2–3) SSRF) | EQ-5D-5L Index 0.77 (0.26) Usual care vs 0.69 (0.32) SSRF; VAS 77 (20) vs 74 (23) | Not provided (only EQ-5D-5L pain dimension 2 (1–3) Usual care vs 2 (2–3) SSRF) | Not provided | Not provided | EQ-5D-5L pain dimension at 6 months 2 (1–3) Usual care vs 2 (2–3) SSRF; time to chest wall pain resolution 54 (25–73) vs 76 (35–104) days | Hospital days median 6 (3–11) Usual care vs 9 (6–20) SSRF | ICU days median 0 (0–1) Usual care vs 0 (0–10) SSRF | Ventilator days 0 (0–0) Usual care vs 0 (0–1) SSRF; tracheostomy 2/42 (5%) vs 7/42 (17%) | 5/42 (12%) Usual care vs 9/42 (21%) SSRF | 0/42 (0%) Usual care vs 0/42 (0%) SSRF (in-hospital) | Not provided (no explicit chronic pain prevalence; only EQ-5D-5L pain scores and time to pain resolution) | Back to work/normal activity: 5/35 (15%) vs 5/37 (15%) at 1 mo; 17/32 (50%) vs 14/34 (40%) at 3 mo; 19/32 (61%) vs 19/36 (58%) at 6 mo | Surgical complications: seroma 1 (2%), SSI 1 (2%) in SSRF; regional analgesia 3 (7%) Usual care vs 7 (17%) SSRF; DVT 0 vs 1, PE 3 vs 1; no overall rate reported | Not provided |
| Prins et al [6] | 2021 | SSRF vs Non-operative management in patients with ≥3 rib fractures or flail chest (overall cohort includes ≥1 rib fracture) | SSRF performed for multiple rib fractures/flail chest according to institutional protocol (plate fixation); detailed implant/approach description not provided | Analgesia per clinical practice; at follow-up 19/300 (6.3%) used daily pain meds (12 NSAID/acetaminophen, 7 opioids) | Not provided (single long-term visit at median 39 months after trauma) | Not provided (only long-term NRS at median 39 months) | Not provided (only long-term SF-12 and EQ-5D at median 39 months) | Not provided | Not provided (EQ-5D and SF-12 only measured once at median 39 months) | Not provided | Moderate–severe thoracic pain (NRS>3 in any of 5 activities) 11/34 (32.4%) SSRF vs 45/214 (21.0%) Non-operative at median 39 months; overall 64/300 (21.3%) | Overall HLOS median 10 (6–20) days; 7 (5–13) for 1–2 ribs, 10 (6–18) for ≥3 ribs, 19 (12–25) for flail chest; SSRF group had longer LOS than Non-operative, exact values not given in main text | ICU admission 113/300 (37.7%); ICU LOS median 6 (3–11) days overall; ICU LOS by treatment not reported | 73/300 (24.3%) required MV overall; MV more frequent with higher injury severity; MV use by treatment reported only qualitatively (higher in SSRF group) | Pneumonia 29/300 (9.7%); part of thoracic complications 49/300 (16.3%) | Not explicitly reported in article (no in-hospital or long-term mortality numbers) | Thoracic pain NRS>0 in 116/300 (38.7%); moderate–severe pain NRS>3 in 64/300 (21.3%); 32.4% SSRF vs 21.0% Non-operative with ≥3 ribs or flail chest | Not specifically reported (no explicit return-to-work or ADL scale; only SF-12 and EQ-5D scores) | Thoracic complications 49/300 (16.3%); surgical reinterventions 28/300 (9.3%); among 35 SSRF patients, implant-related issues in 6 (18%) and hardware removal in 6 (17.1%) | Symptomatic nonunion 1/300 (0.3%); chest wall deformity otherwise not detailed |
| Walters et al [33] | 2019 | Surgical rib fixation with locking plates (MatrixRIB system) vs nonoperative management for flail chest injuries | Low-profile precontoured anatomical titanium locking plates (MatrixRIB) via muscle-sparing lung-up thoracotomy; enough ribs (mostly 3rd–9th) fixed to stabilize chest wall | Standardized chest trauma pathway: paracetamol, NSAIDs, oral morphine as needed, neuraxial/epidural analgesia or mechanical ventilation when indicated, daily specialist physiotherapy, incentive spirometry, low-molecular-weight heparin and supportive care | Not provided (patient-reported outcomes collected at mean ~18–21 months only) | Not provided | Not provided | Not provided | EQ-5D-5L index: 0.603 (SD 0.31, median 0.6885, n=36) surgical vs 0.604 (SD 0.312, median 0.6640, n=25) nonoperative at mean follow-up 17.6 vs 20.9 months | Mean pain VAS (0–10) at follow-up: 3.41 (SD 2.9, median 3.5) surgical vs 3.52 (SD 3.4, median 2) nonoperative | Single long-term assessment (mean 17.6 vs 20.9 months) showed similar pain VAS between groups (no significant difference, P=0.966) | HLOS 31.0 (SD 35.3, median 19, range 4–172) surgical vs 13.0 (SD 16.4, median 9, range 1–123) nonoperative; after excluding deaths/outliers 21.9 (SD 14.3, median 18) vs 11.8 (SD 8.2, median 9) | ICU admission 89.6% surgical vs 43.8% nonoperative; ICU LOS 11.6 (SD 9.8, median 10.5, range 0–46) surgical vs 4.9 (SD 8.5, range 0–48) nonoperative | Not explicitly reported (all managed on standardized pathway with MV/epidural as needed; MV duration not given by group) | Not explicitly reported by group (complications not broken down in tables) | 30-day mortality 1/56 (1.8%) surgical vs 11/89 (12.4%) nonoperative; after excluding two head-injury deaths 1/56 (1.8%) vs 9/87 (10.3%) | Not provided as prevalence; only mean long-term pain VAS scores (3.41 vs 3.52) | EQ-5D-5L domains, perceived health (VAS), and UCLA Activity Score changes similar between groups; no significant differences or clear return-to-work percentages | Not summarized as overall rate; length of stay and ICU use higher in surgical group but likely reflect higher injury severity; specific complications not quantified | Not reported (no explicit chest wall deformity or nonunion rates) |
| Xu et al [34] | 2025 | Surgical treatment vs conservative treatment for non-flail multiple rib fractures | Open reduction and internal fixation with titanium rib fixation plates (Waston Medical); muscle-sparing thoracotomy, fixation arms bilaterally over each fracture, postoperative thoracostomy drainage and early ambulation | Standardized perioperative care: prophylactic cephalosporins (cefazolin 1 g q8h), multimodal analgesia, oxygen therapy, chest drainage for hemopneumothorax, early mobilization and pulmonary physiotherapy | Quality of life index 0.78 surgical vs 0.47 conservative at 3 months (scale 0–1, instrument not explicitly named) | Not explicitly reported at 3 months; early VAS in hospital: 3.68 ± 0.88 vs 2.76 ± 0.84 at day 2, 1.67 ± 0.68 vs 1.36 ± 0.39 at day 4 | Quality of life index 0.98 surgical vs 0.64 conservative at 6 months | Not reported as mean NRS; chronic pain at follow-up categorized by VAS grades (see Chronic Pain%) | Not provided (follow-up reported to 6 months only) | Not provided | At 6-month follow-up, VAS categories: painless 77.1% vs 66.7%, mild 16.4% vs 20.6%, moderate 6.6% vs 12.7%, severe 0% vs 0% (surgical vs conservative) | 21.32 ± 2.56 days surgical vs 18.74 ± 3.62 days conservative | Not explicitly reported | Not explicitly reported (mechanical ventilation initiated for PaO₂/FiO₂ <200 mm Hg, but no group-level duration or counts) | Pulmonary infection 6/61 (9.8%) surgical vs 14/63 (21.6%) conservative (P = .011) | Not reported (no deaths described) | At 6 months, moderate pain (VAS 4–6) 6.6% surgical vs 12.7% conservative; no severe pain in either group | Psychological recovery better with surgery: HAMA 21.64 ± 3.36 vs 32.36 ± 5.41; HAMD 28.74 ± 4.14 vs 38.62 ± 4.37 after treatment; lung function (PaO₂, PaCO₂, PaO₂/FiO₂) normalized faster in surgical group | During hospitalization: pleural effusion 18.0% vs 20.6%, delayed hemothorax 4.9% vs 3.1%, pulmonary infection 9.8% vs 21.6% (significantly lower with surgery), atelectasis 3.3% vs 6.3%, displaced rib 1.6% vs 3.2% | Not reported |
| Zhang et al[35] | 2019 | Surgical treatment (titanium plate fixation) vs conservative treatment for severe non-flail chest rib fractures | Open reduction and internal fixation with titanium plates and screws; mean 4.74 ribs fixed, small incisions (~7.7 cm), chest wall and thoracic drainage with early mobilization | Conservative: stepwise analgesia starting with NSAIDs, escalating to opioids; some received thoracic epidural, paravertebral or intercostal nerve blocks; both groups had standard symptomatic and supportive care | SF-36 total score 393.57 ± 68.51 surgical vs 335.12 ± 83.11 conservative | 0.90 ± 0.45 surgical vs 2.92 ± 0.27 conservative | SF-36 total score 493.07 ± 69.97 surgical vs 363.64 ± 72.02 conservative | 0.36 ± 0.49 surgical vs 2.05 ± 0.65 conservative | Not provided (follow-up reported to 6 months) | Not provided | At 6 months NRS 0.36 ± 0.49 surgical vs 2.05 ± 0.65 conservative (sustained pain relief with surgery) | Not provided | Not provided | Not quantified by group (MV sometimes required but no numbers reported) | Not reported by group | No perioperative deaths reported in either group | Not reported as prevalence; chronic pain described using NRS means at 3 and 6 months | Daily physical activity higher in surgery group at 3 months (27/39 vs 19/39); return to pre-injury work at 6 months 94.9% (37/39) surgical vs 64.1% (25/39) conservative | Surgery group: lower-extremity DVT 2/39 and minor soft-tissue issues in 3; no infections or hardware failures; overall complication rate not summarized, conservative group complications not quantified | No thoracic deformity, rib plate fracture, or bone nonunion observed on follow-up imaging in any patient |
| HRQoL, health-related quality of life; NRS, numeric rating scale; ICU, intensive care unit. Hospitalization days and ICU stay days are reported as in the original publications (mean ± SD or median [IQR]). Chronic pain (%) refers to the proportion of patients with persistent chest wall pain at ≥3 months of follow-up. Blank cells indicate that the corresponding data were not reported. | | | | | | | | | | | | | | | | | | | | |
