# Supplementary figures and images for: Long-term quality of life and chronic pain after surgical vs. non-operative treatment of rib fractures: systematic review and meta-analysis
Source: Front Surg. 2026 Mar 30;13:1774082. doi: 10.3389/fsurg.2026.1774082 (PMC13070923; doi:10.3389/fsurg.2026.1774082)

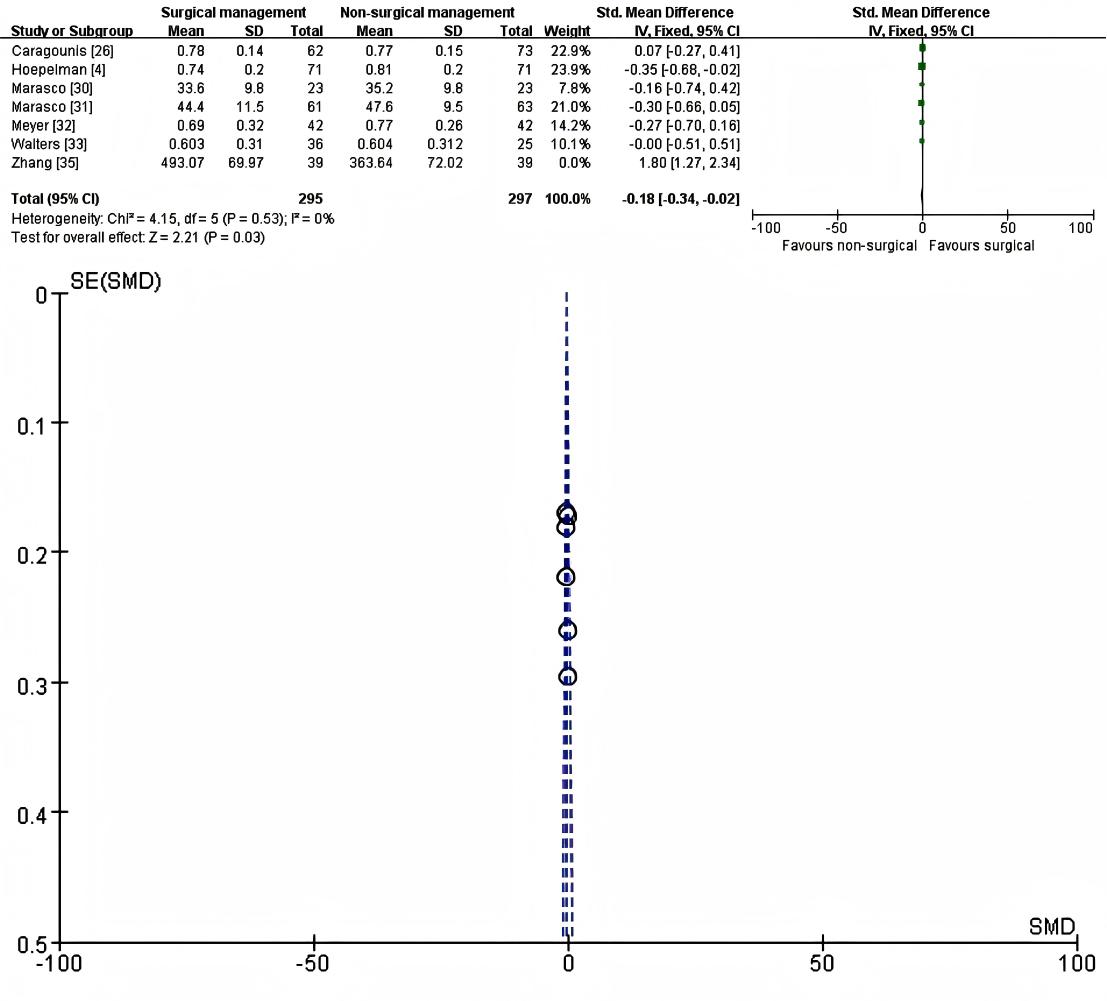

Supplement: Supplementary Figure S1 — Sensitivity analysis of HRQoL comparing SSRF with non-operative management using a fixed-effect model, shown as a forest plot (left) with the corresponding Galbraith plot (right). HRQoL, health-related quality of life; SSRF, surgical stabilisation of rib fractures; SMD, standardized mean difference; CI, confidence interval; SE, standard error. [file Supplementaryfile1.zip › Image 1.jpg]

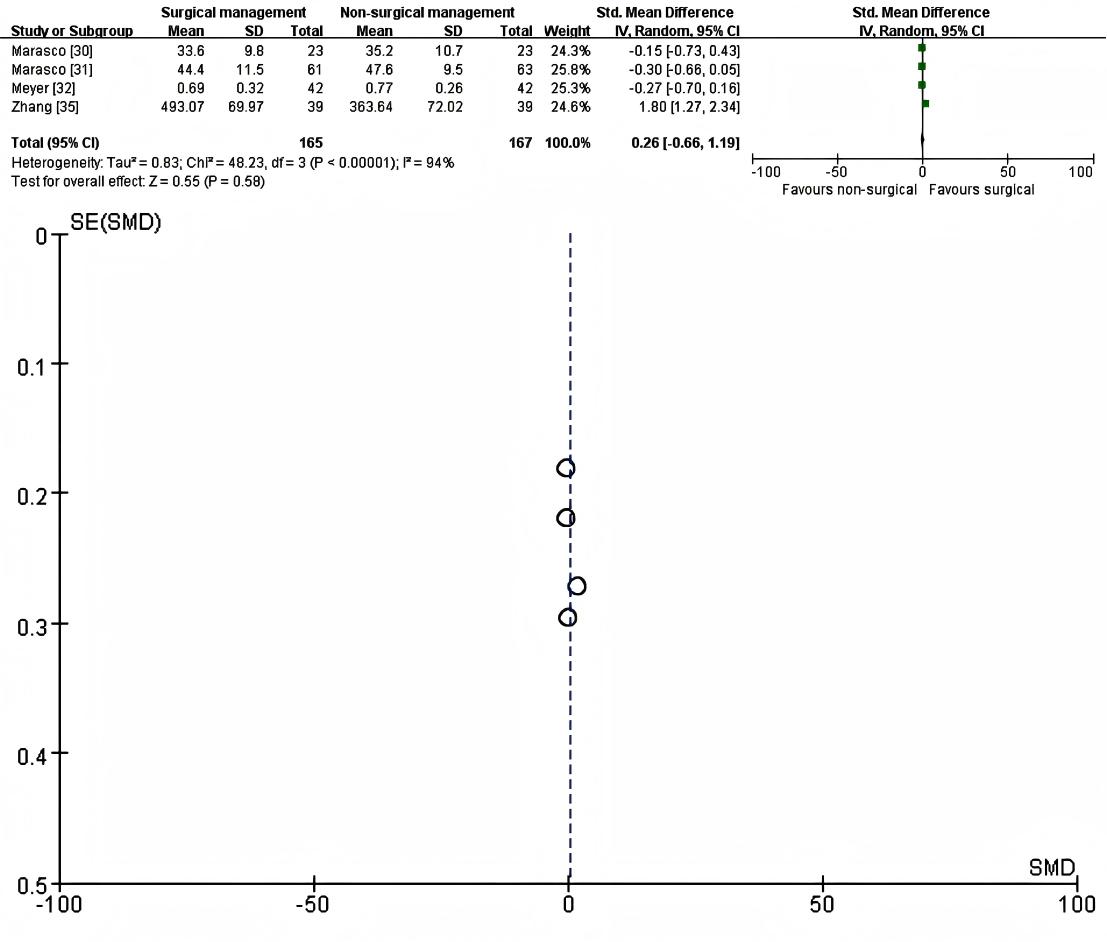

Supplement: Supplementary Figure S1 — Sensitivity analysis of HRQoL comparing SSRF with non-operative management using a fixed-effect model, shown as a forest plot (left) with the corresponding Galbraith plot (right). HRQoL, health-related quality of life; SSRF, surgical stabilisation of rib fractures; SMD, standardized mean difference; CI, confidence interval; SE, standard error. [file Supplementaryfile1.zip › Image 2.jpg]

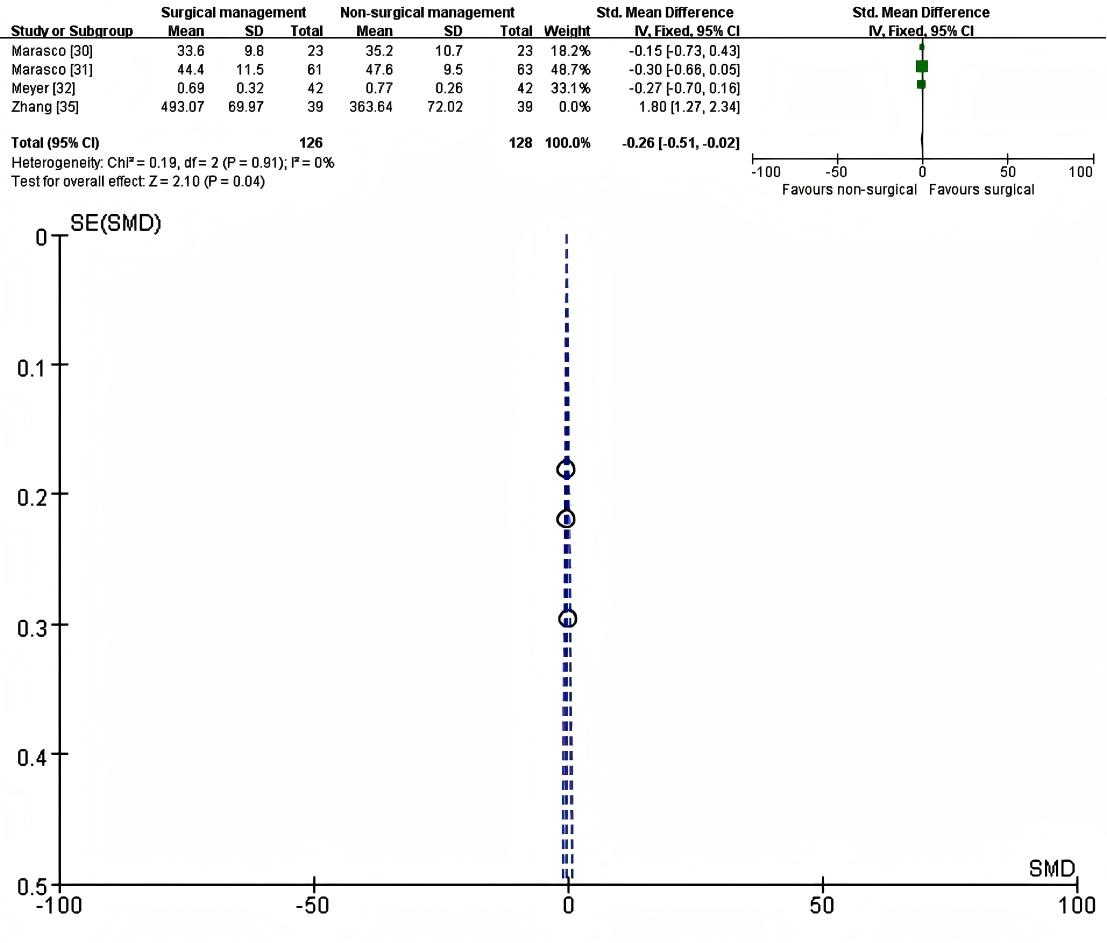

Supplement: Supplementary Figure S1 — Sensitivity analysis of HRQoL comparing SSRF with non-operative management using a fixed-effect model, shown as a forest plot (left) with the corresponding Galbraith plot (right). HRQoL, health-related quality of life; SSRF, surgical stabilisation of rib fractures; SMD, standardized mean difference; CI, confidence interval; SE, standard error. [file Supplementaryfile1.zip › Image 3.jpg]

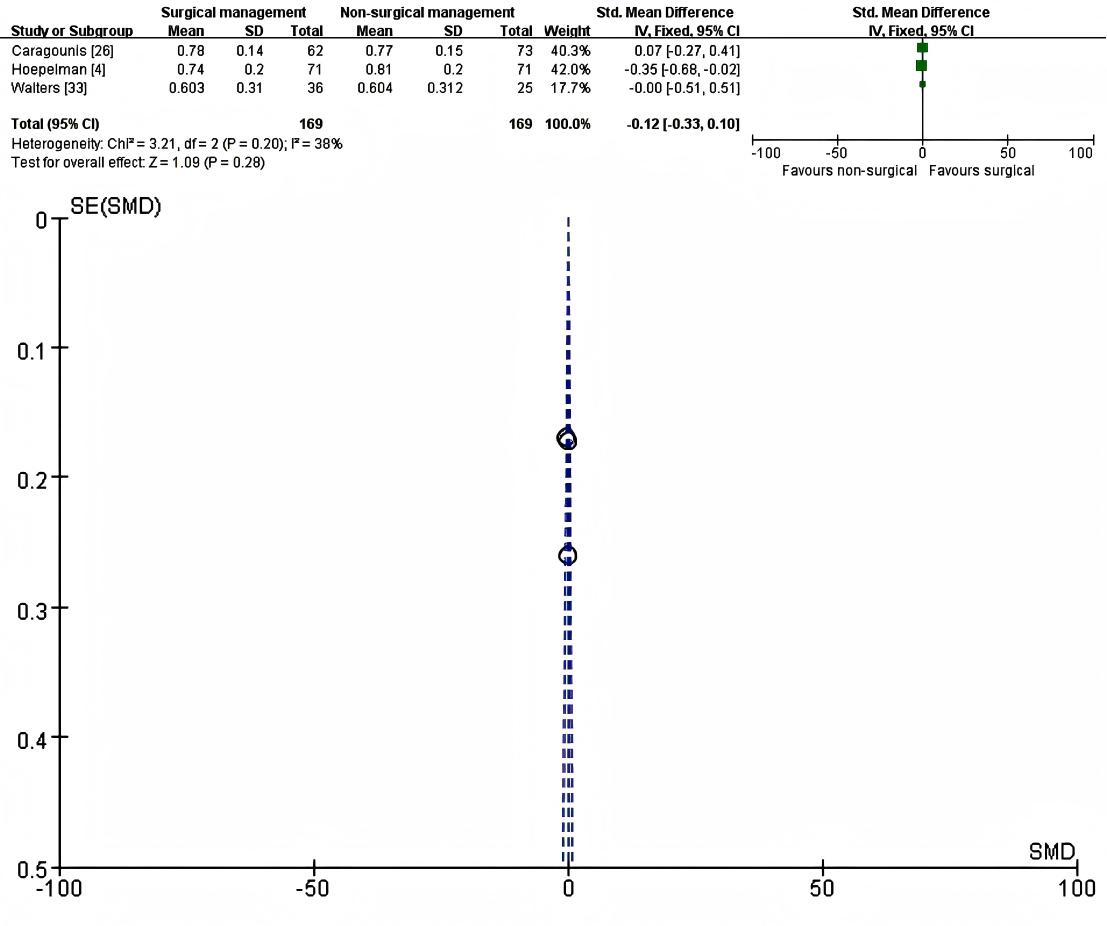

Supplement: Supplementary Figure S1 — Sensitivity analysis of HRQoL comparing SSRF with non-operative management using a fixed-effect model, shown as a forest plot (left) with the corresponding Galbraith plot (right). HRQoL, health-related quality of life; SSRF, surgical stabilisation of rib fractures; SMD, standardized mean difference; CI, confidence interval; SE, standard error. [file Supplementaryfile1.zip › Image 4.jpg]

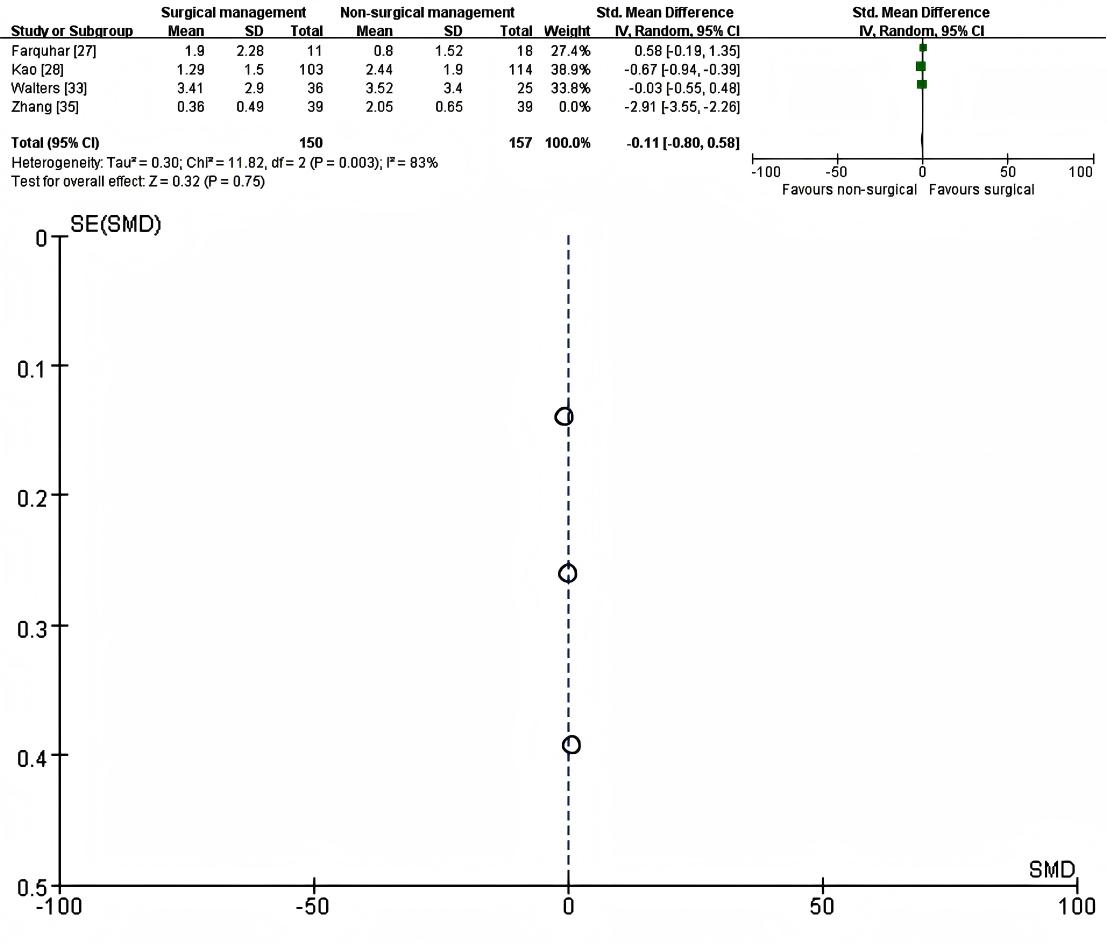

Supplement: Supplementary Figure S1 — Sensitivity analysis of HRQoL comparing SSRF with non-operative management using a fixed-effect model, shown as a forest plot (left) with the corresponding Galbraith plot (right). HRQoL, health-related quality of life; SSRF, surgical stabilisation of rib fractures; SMD, standardized mean difference; CI, confidence interval; SE, standard error. [file Supplementaryfile1.zip › Image 5.jpg]
